# Manipulation of IRE1-Dependent MAPK Signaling by a Vibrio Agonist-Antagonist Effector Pair

**DOI:** 10.1128/mSystems.00872-20

**Published:** 2021-02-09

**Authors:** Nicole J. De Nisco, Amanda K. Casey, Mohammed Kanchwala, Alexander E. Lafrance, Fatma S. Coskun, Lisa N. Kinch, Nick V. Grishin, Chao Xing, Kim Orth

**Affiliations:** a Department of Molecular Biology, University of Texas Southwestern Medical Center, Dallas, Texas, USA; b Howard Hughes Medical Institute, University of Texas Southwestern Medical Center, Dallas, Texas, USA; c Department of Biological Sciences, University of Texas at Dallas, Richardson, Texas, USA; d McDermott Center for Human Growth and Development, University of Texas Southwestern Medical Center, Dallas, Texas, USA; e Department of Immunology, University of Texas Southwestern Medical Center, Dallas, Texas, USA; f Department of Biophysics and Biochemistry, University of Texas Southwestern Medical Center, Dallas, Texas, USA; g Department of Bioinformatics, University of Texas Southwestern Medical Center, Dallas, Texas, USA; h Department of Population and Data Sciences, University of Texas Southwestern Medical Center, Dallas, Texas, USA; University of Wisconsin-Madison

**Keywords:** Erk, Ire1, T3SS, UPR, V-ATPase, *Vibrio parahaemolyticus*, effector

## Abstract

Diverse bacterial pathogens employ effector delivery systems to disrupt vital cellular processes in the host (N. M. Alto and K. Orth, Cold Spring Harbor Perspect Biol 4:a006114, 2012, https://doi.org/10.1101/cshperspect.a006114). The type III secretion system 1 of the marine pathogen Vibrio parahaemolyticus utilizes the sequential action of four effectors to induce a rapid, proinflammatory cell death uniquely characterized by a prosurvival host transcriptional response (D. L. Burdette, M. L. Yarbrough, A Orvedahl, C. J. Gilpin, and K. Orth, Proc Natl Acad Sci USA 105:12497–12502, 2008, https://doi.org/10.1073/pnas.0802773105; N. J. De Nisco, M. Kanchwala, P. Li, J. Fernandez, C. Xing, and K. Orth, Sci Signal 10:eaa14501, 2017, https://doi.org/10.1126/scisignal.aal4501). Herein, we show that this prosurvival response is caused by the action of the channel-forming effector VopQ that targets the host V-ATPase, resulting in lysosomal deacidification and inhibition of lysosome-autophagosome fusion. Recent structural studies have shown how VopQ interacts with the V-ATPase and, while in the ER, a V-ATPase assembly intermediate can interact with VopQ, causing a disruption in membrane integrity. Additionally, we observed that VopQ-mediated disruption of the V-ATPase activates the IRE1 branch of the unfolded protein response (UPR), resulting in an IRE1-dependent activation of ERK1/2 MAPK signaling. We also find that this early VopQ-dependent induction of ERK1/2 phosphorylation is terminated by the VopS-mediated inhibitory AMPylation of Rho GTPase signaling. Since VopS dampens VopQ-induced IRE1-dependent ERK1/2 activation, we propose that IRE1 activates ERK1/2 phosphorylation at or above the level of Rho GTPases. This study illustrates how temporally induced effectors can work as in tandem as agonist/antagonist to manipulate host signaling and reveals new connections between V-ATPase function, UPR, and MAPK signaling.

**IMPORTANCE** Vibrio parahaemolyticus is a seafood-borne pathogen that encodes two type 3 secretion systems (T3SS). The first system, T3SS1, is thought to be maintained in all strains of V. parahaemolyticus to maintain survival in the environment, whereas the second system, T3SS2, is linked to clinical isolates and disease in humans. Here, we found that first system targets evolutionarily conserved signaling systems to manipulate host cells, eventually causing a rapid, orchestrated cells death within 3 h. We have found that the T3SS1 injects virulence factors that temporally manipulate host signaling. Within the first hour of infection, the effector VopQ acts first by activating host survival signals while diminishing the host cell apoptotic machinery. Less than an hour later, another effector, VopS, reverses activation and inhibition of these signaling systems, ultimately leading to death of the host cell. This work provides example of how pathogens have evolved to manipulate the interplay between T3SS effectors to regulate host signaling pathways.

## Abstract (precis)

Vibrio parahaemolyticus is a seafood-borne pathogen that encodes two type 3 secretion systems (T3SS). The first system, T3SS1, is thought to be maintained in all strains of V. parahaemolyticus to maintain survival in the environment, whereas the second system, T3SS2, is linked to clinical isolates and disease in humans.

## INTRODUCTION

Diverse bacterial pathogens employ effector delivery systems to disrupt vital cellular processes in the host (1). The seafood-borne pathogen Vibrio parahaemolyticus uses two needle-like type III secretion systems (T3SS1 and T3SS2) to inject effectors into host cells to manipulate signaling and cellular processes during infection (2). The V. parahaemolyticus T3SS2 is found in clinical isolates, is linked to disease in humans, and has been shown to mediate invasion of mammalian host cells (3, 4). In contrast, T3SS1 is present in all V. parahaemolyticus isolates and is thus believed to be essential for survival in its environmental niche. This niche has been rapidly expanding due to the warming of coastal waters, contributing to the resurgence of V. parahaemolyticus as a significant cause of gastroenteritis worldwide (5, 6). Together, the V. parahaemolyticus T3SS1 effectors orchestrate a temporally regulated nonapoptotic cell death in cultured cells (2). The specific cell type that T3SS1 has evolved to target in the environment remains undefined; however, its effectors target processes that are conserved from yeasts to humans (2, 7–9).

**FIG 1:**
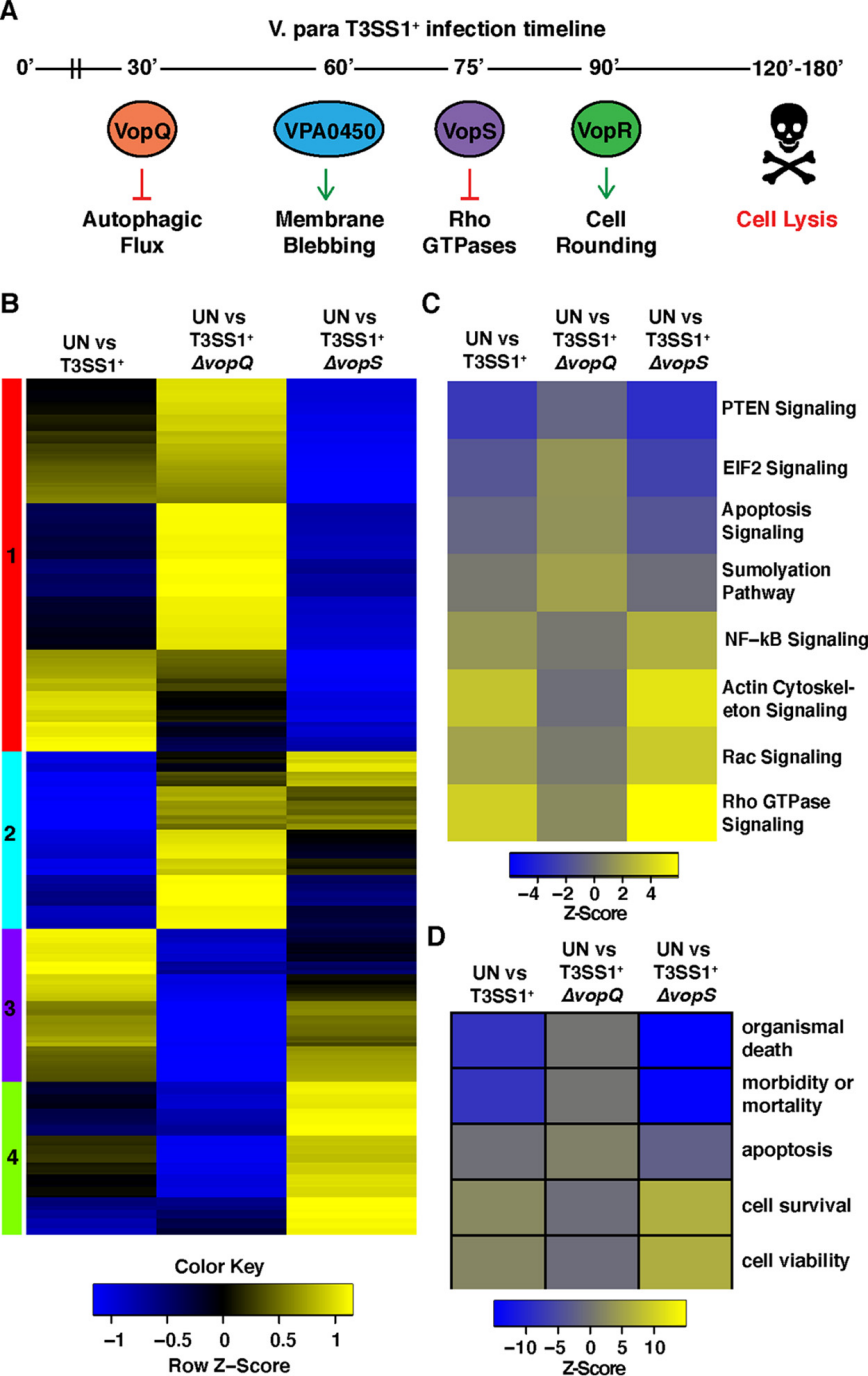
VopQ and VopS have antagonistic effect on T3SS1-specific pathway and network induction. (A) Illustration of temporal effector function during T3SS1-mediated cell death. (B) Heat map of normalized differential expression of previously identified T3SS1-specific transcripts in uninfected (UN) primary human fibroblasts compared to primary human fibroblasts infected with either V. parahaemolyticus T3SS1^+^, T3SS1^+^Δ*vopQ*, or T3SS1^+^Δ*vopS* for 90 min. Yellow denotes transcripts with relative increased abundance infected cells compared to UN cells, and blue denotes decreased abundance. Clusters (color bars on the left) were assigned through hierarchical clustering of the differential expression data. (C) Heat map of predicted repression (blue) and activation (yellow) Z-scores calculated from differential expression data for UN versus V. parahaemolyticus T3SS1^+^, UN versus V. parahaemolyticus T3SS1^+^Δ*vopQ*, and UN versus V. parahaemolyticus T3SS1^+^Δ*vopS* using Qiagen’s Ingenuity Pathway Analysis software. The color key correlates the displayed heat map color and calculated Z-scores, and gray denotes unaffected (*P > *0.05) pathways. (D) Heat map of Ingenuity Pathway Analysis Z-score prediction of repression (blue) or activation (yellow) of biological networks after 90 min of POR3:T3SS1^+^, T3SS1^+^Δ*vopQ*, and T3SS1^+^Δ*vopS* infection.

Cell death mediated by T3SS1 occurs in distinct and highly reproducible stages through the temporal action of four known effectors: VopQ (VP1680), VPA0450, VopS (VP1686), and VopR (VP1683) (Fig. 1A) (2, 10). Within 30 min of a synchronized infection, VopQ forms an outward-rectifying channel in V-ATPase-containing membranes, resulting in neutralization of the compartment (e.g., vacuole or lysosome) and inhibition of membrane fusion (Fig. 1A) (8, 11). These two activities inhibit autophagic flux, resulting in massive autophagosome accumulation, and contribute to a proinflammatory cell death within 3 h (12–14). Interestingly, recent work has demonstrated that VopQ also induces metabolic changes within Caco-2 cells, specifically altering glycolysis, the citric acid cycle, and amino acid metabolism (15). VPA0450 is a phosphatidyl 5-phosphatase that hydrolyzes phosphatidylinositol 4,5-bisphosphate [PI(4,5)P_2_] at about 1 h after infection, resulting in blebbing of the plasma membrane (Fig. 1A) (16). Soon after VPA0450-mediated blebbing is observed, VopS, a Fic (filamentation induced by cAMP) domain-containing protein, covalently attaches an AMP to a threonine residue in the switch 1 region of Rho GTPases Rho, Rac, and Cdc42. This modification, termed AMPylation, inactivates the Rho GTPases, thereby precipitating cytoskeletal collapse and cell rounding, as well as inactivation of nuclear factor kappa B (NF-κB) and mitogen-activated protein kinase (MAPK) signaling pathways (Fig. 1A) (9, 17). The fourth effector, VopR, causes cell rounding around 90 min postinfection, but its activity has remained elusive (10).

Despite the rapid, nonapoptotic cell death orchestrated by T3SS1, our previous studies (18) have uncovered evidence that the T3SS1 rewires host gene expression to subvert cell death and activate cell survival pathways, including MAPK signaling pathways. We performed a systems-level analysis of the temporal changes in host cell gene expression during V. parahaemolyticus infection to understand how the T3SS1 effectors work in concert to orchestrate cell death and subvert host immune responses. The host transcriptional response to T3SS1 resulted in the activation of host cell survival networks and repression of cell death networks (18).

Previously, it was found that VopQ was both necessary and sufficient for the accumulation of LC3-positive autophagosomes as well as the deacidification of endolysosomal compartments. These effects are caused by direct interaction between VopQ and the V_o_ subcomplex of the V-ATPase. Recently, cryo-EM studies performed in our lab revealed extensive interactions between VopQ and the c subunit of V_o_ V-ATPase that stabilize the insertion of VopQ in the membrane alongside the V-ATPase (19). The interactions of VopQ with the c ring of V_o_ is predicted to form an unconventional membrane pore through the juxtaposition of charged resides of VopQ against the hydrophobic lipid environment (19). This disruption is predicted to lead to the deacidification of the lysosomal membrane. We found that a fully functioning or assembled V-ATPase at the vacuole is not necessary to induce VopQ toxicity in Saccharomyces cerevisiae and that VopQ can interact with an assembly intermediate of the V-ATPase (V_o_ c ring) in the endoplasmic reticulum (ER), resulting in cell death (19). As VopQ forms a pore in target membranes, the ER membrane is compromised, and this could lead to the induction of host cell signaling events, including the unfolded protein response (UPR).

Here, we found that VopQ activates the inositol-requiring enzyme 1 (IRE1) branch of the UPR in yeast and cultured cells. We demonstrate that the activation of IRE1 by VopQ results in an induction of extracellular signal-regulated protein kinase 1/2 (ERK1/2) signaling that is dependent on IRE1 kinase but not nuclease activity. We also found that another T3SS1 effector, VopS, dampens VopQ-mediated activation of ERK1/2 signaling by AMPylation-dependent inactivation of Rho GTPases, thereby limiting the activation of ERK1/2 signaling to early infection time points. Taken together, our results provide another example of the interplay between T3SS effectors and how they can temporally regulate host signaling pathways.

## RESULTS

### VopQ and VopS have antagonistic effects on T3SS1-specific pathway and network induction.

Previously, we discovered that T3SS1 activates host cell survival networks and represses cell death networks (18). Since autophagy is linked to prosurvival network signaling, our findings led us to ask if VopQ could be responsible for prosurvival signals observed during infection. To understand the contribution of individual effectors to the T3SS1-specific transcriptional response, we characterized infection of primary human dermal fibroblasts (PHDFs) with V. parahaemolyticus strains carrying deletions of either *vopQ* or *vopS.* We chose this cell line so that our data would be comparable with the data obtained in our previous transcriptomic analysis and because, being primary cells, PHDFs do not carry transforming mutations that could alter cell signaling pathways (18). We included VopS because it targets Rho GTPases that regulate MAPK signaling (9, 17). We used V. parahaemolyticus strain POR3, a derivative of the clinical strain RIMD2210633 that does not produce functional hemolysins or a functional T3SS2 (Δ*tdhAS* Δ*vcrD2*) but maintains an active T3SS1 (5). This strain and its Δ*vopQ* and Δ*vopS* derivatives are referred to herein as T3SS1^+^, T3SS1^+^Δ*vopQ*, and T3SS1^+^Δ*vopS*, respectively (see Table S1 in the supplemental material). As observed with previously characterized cell types, cytotoxicity of PHDFs occurring within the first 4 h of infection was completely dependent on VopQ and independent of VopS (see Fig. S1A and B) (2). We then performed RNA sequencing on the PHDFs after 90 min of infection with V. parahaemolyticus T3SS1^+^, T3SS1^+^Δ*vopQ*, and T3SS1^+^Δ*vopS*. The sequencing data passed statistical quality control tests, and principal-component analysis indicated tight clustering of replicates (Fig. S2A). Complete differential expression data are reported in Data Set S1, sheet 1, but for this study, we focused on the 398 host genes previously found to be differentially expressed specifically in response to T3SS1 (18).

10.1128/mSystems.00872-20.1 FIG S1 Rapid cytotoxicity of V. parahaemolyticus T3SS1 is dependent on the effector VopQ. Lactate dehydrogenase (LDH) released by primary human dermal fibroblasts infected with (a) V. parahaemolyticus T3SS1^−^, T3SS1^+^, T3SS1^+^Δ*vopQ*, and T3SS1^+^Δ*vopQ*+p*vopQ* or (b) V. parahaemolyticus T3SS1^−^, T3SS1^+^, T3SS1^+^Δ*vopS*, and T3SS1^+^Δ*vopS*+p*vopS*. Percent cytotoxicity was calculated relative to uninfected (negative) and Triton X-100-treated (positive) controls. Error bars show standard deviations (SD), and data are means for 9 replicates (3 biological replicates and 3 technical replicates). *P* values were calculated by one-way ANOVA and Dunnett’s multiple-comparison test (***, *P < *0.001; ****, *P < *0.0001; n.s., not significant). Download FIG S1, TIF file, 0.3 MB. Copyright © 2021 De Nisco et al. 2021 De Nisco et al. This content is distributed under the terms of the Creative Commons Attribution 4.0 International license.

10.1128/mSystems.00872-20.2 FIG S2 Analysis of RNA sequencing data and RT-qPCR verification. (a) Data for each sample plotted on a two-dimensional scatterplot so that distances on the plot approximate the expression differences between the samples. BCV, biological coefficient of variation. Minimal variation was observed between replicates. (b) Venn diagram depicting overlap of the differential expression of previously identified T3SS1-specific genes between comparisons. (c and d) Relative expression of *EGR1* (c) and *FOS* (d) in primary human dermal fibroblasts infected with V. parahaemolyticus T3SS1^−^, T3SS1^+^ and T3SS1^+^-derivative strains with respect to uninfected cells, measured by quantitative RT-PCR. Expression was normalized to the housekeeping gene *IPO8*. Data are 2^−ΔΔ^*^Cq^* ± SEM from three independent experiments. *P* values were calculated by one-way ANOVA and Dunnett’s multiple-comparison test (**, *P < *0.01; ***, *P < *0.001; ****, *P < *0.0001). Download FIG S2, TIF file, 0.6 MB. Copyright © 2021 De Nisco et al. 2021 De Nisco et al. This content is distributed under the terms of the Creative Commons Attribution 4.0 International license.

10.1128/mSystems.00872-20.7 DATA SET S1 Complete RNA sequencing data. Sheet 1, complete RNA sequencing differential expression data. Sheet 2, differential expression of T3SS1-specific genes. Sheet 3, IPA canonical pathway analysis of differential expression data. Sheet 4, IPA network (disease and biofunction) analysis of differential expression data. Download Data Set S1, XLSX file, 10.9 MB. Copyright © 2021 De Nisco et al. 2021 De Nisco et al. This content is distributed under the terms of the Creative Commons Attribution 4.0 International license.

10.1128/mSystems.00872-20.8 TABLE S1 Bacterial strains used in this work. Download Table S1, DOCX file, 0.02 MB. Copyright © 2021 De Nisco et al. 2021 De Nisco et al. This content is distributed under the terms of the Creative Commons Attribution 4.0 International license.

The hierarchically clustered expression heat map in Fig. 1B illustrates how T3SS1 causes changes in expression of these 398 genes in the absence of either VopQ or VopS. Of these T3SS1-specific genes, 146 were similarly differentially expressed in the uninfected (UN) versus T3SS1^+^-infected and UN versus T3SS1^+^Δ*vopQ*-infected cells, and 197 were similarly differentially expressed in the UN versus T3SS1^+^-infected and UN versus T3SS1^+^Δ*vopS*-infected cells (Fig. S2B; Data Set S1, sheet 2). Two hundred fifty-two and 201 T3SS1-specific genes either were not differentially expressed or changed direction during T3SS1^+^Δ*vopQ* and T3SS1^+^Δ*vopS* infection, respectively. Expression of many genes, especially those within clusters 1 and 4, was oppositely affected during infection with T3SS1^+^Δ*vopQ* compared to during infection with T3SS1^+^Δ*vopS* (Fig. 1B; Data Set S1, sheet 2). Notably, expression of the *EGR1* and *FOS* transcription factors, which are known to be regulated by MAPK signaling pathways, was reduced in T3SS1^+^Δ*vopQ*-infected cells compared to T3SS1^+^-infected cells and highly elevated by T3SS1^+^Δ*vopS* infection (Data Set S1, sheet 2) (20). We validated these findings by quantitative reverse transcription-PCR (RT-PCR) and by using V. parahaemolyticus strains with deletions of multiple effectors (T3SS1^+^ Δ*vopQR* Δ*vpa0450* and T3SS1^+^ Δ*vopRS* Δ*vpa0450*) and showed that VopQ is necessary and sufficient for the elevated expression of both *FOS* and *EGR1* (Fig. S2C and D).

We next used Ingenuity Pathway Analysis (IPA) to understand how the activities of VopQ and VopS contribute to the changes in host signaling events induced by the T3SS1 (Data Set S1, sheet 3). The T3SS1-specific induction or repression of many pathways was dependent on VopQ and enhanced in the absence of VopS (Fig. 1C). For example, induction of NF-κB signaling, actin cytoskeleton signaling, and Rho GTPase signaling by T3SS1 was greatly reduced in the absence of VopQ and enhanced in the absence of VopS. These observations are consistent with the opposing effects on differential expression patterns observed in Fig. 1B.

### VopQ induces prosurvival signaling networks.

To understand the relative contributions of VopQ and VopS to the host response to T3SS1 on the network level, we used IPA to perform biological function network analysis. Previously, we had shown that the T3SS1 activates cell survival networks and represses cell death networks (18). Strikingly, this effect was completely lost during T3SS1^+^Δ*vopQ* infection and amplified during T3SS1^+^Δ*vopS* infection (Fig. 1D; Data Set S1, sheet 4). Specifically, we observed a loss in cell survival, viability signaling network activation, and death and mortality signaling network repression in PHDFs infected with V. parahaemolyticus T3SS1^+^Δ*vopQ* compared to V. parahaemolyticus T3SS1^+^, while infection with T3SS1^+^Δ*vopS* instead amplified these signaling changes (Fig. 1D). The apoptosis signaling network, which is normally repressed during T3SS1^+^ infection, was activated during infection with T3SS1^+^Δ*vopQ* (Fig. 1D). These data support a model in which the activity of VopQ elicits transcriptional changes in the host cell that result in the activation of cell survival and repression of cell death networks, and VopS may function to dampen this response.

### VopQ induces a pulse of ERK1/2 signaling that is dampened by VopS.

To further dissect VopQ’s effect on host signaling pathways in mammalian cells, we continued with a more genetically tractable model, mouse embryonic fibroblasts (MEFs). We characterized the cytotoxicity of the V. parahaemolyticus T3SS1^+^ strain and its derivates in MEFs. The cell death induced by T3SS1 occurred over a similar time scale in MEFs as in PHDFs and was similarly dependent on VopQ (Fig. S3A). This result was expected, because T3SS1-mediated cell death is conserved across diverse cell types (2, 8, 17, 18). We chose to examine the ERK1/2 signaling pathway because the RNA sequencing data suggested that VopQ activates Rho GTPase signaling (Fig. 1C), and in previous work we demonstrated that T3SS1-induced *EGR1* and *FOS* expression requires active mitogen-activated protein kinase kinases 1 and 2 (MEK1/2), the kinases upstream of ERK1/2 (18). Furthermore, as we did not observe agonist and antagonist effects of VopQ and VopS, respectively, on the expression of c-Jun N-terminal kinase (JNK) signaling target genes, we focused our analysis on the ERK1/2 pathway (Table S2).

10.1128/mSystems.00872-20.3 FIG S3 Conservation of T3SS1 VopQ-dependent cytotoxicity and MEK1/2 dependent EGR1 expression in MEFs. (a) Lactate dehydrogenase (LDH) released by MEFs infected with V. parahaemolyticus T3SS1^−^, T3SS1^+^, T3SS1^+^Δ*vopQ*, T3SS1^+^Δ*vopS*, or T3SS1^+^*vopQ*^S200P^ strains. Percent cytotoxicity was calculated relative to uninfected (negative) and Triton X-100-treated (positive) controls. Error bars show SD, and data are means for 9 replicates (3 biological replicates and 3 technical replicates). (b) RT-qPCR showing relative expression of *Egr1* in MEFs pretreated with MEK1/2 inhibitor U0126 or DMSO (vehicle) and then infected with V. parahaemolyticus T3SS1^−^ or V. parahaemolyticus T3SS1^+^ compared to uninfected cells. Expression was normalized to the housekeeping gene *IPO8*, and data are 2^−ΔΔ^*^Cq^* ± SEM from three independent experiments. *P* values were calculated by one-way ANOVA and Dunnett’s multiple-comparison test (*, *P < *0.02, **; *P < *0.01; ****, *P < *0.0001; n.s., not significant). (c) Immunoblot showing p-Erk1/2 and total Erk1/2 in starved MEFs infected with T3SS1^+^, T3SS1^+^Δ*vopS*, T3SS1^+^Δ*vopS*+p*vopS*, and T3SS1^+^Δ*vopS*+p*vopS^H348A^* V. parahaemolyticus strains for 45, 60, and 75 min. T3SS1^+^Δ*vopS* and T3SS1^+^Δ*vopS*+p*vopS^H348A^* induced prolonged Erk1/2 phosphorylation, which was not observed in T3SS1^+^- or T3SS1^+^Δ*vopS*+p*vopS*-infected MEFs. Download FIG S3, TIF file, 1.2 MB. Copyright © 2021 De Nisco et al. 2021 De Nisco et al. This content is distributed under the terms of the Creative Commons Attribution 4.0 International license.

10.1128/mSystems.00872-20.9 TABLE S2 Expression of alternate MAPK pathway target genes. Download Table S2, DOCX file, 0.01 MB. Copyright © 2021 De Nisco et al. 2021 De Nisco et al. This content is distributed under the terms of the Creative Commons Attribution 4.0 International license.

**FIG 2:**
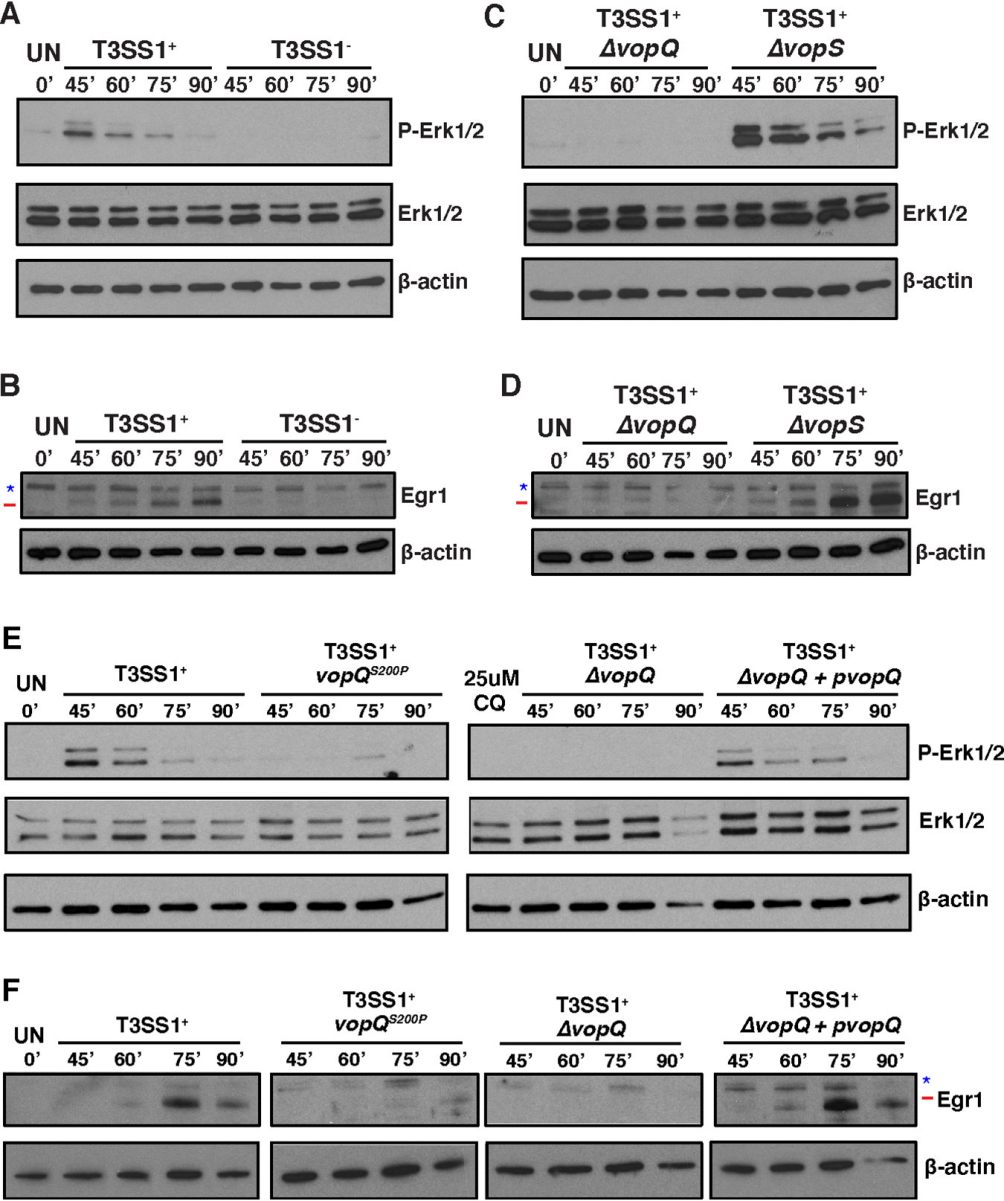
VopQ but not VopQ^S200P^ induces an early activation of ERK1/2 MAPK signaling. (A) Immunoblot showing phosphorylated Erk1 and Erk2 (p-Erk1/2) and total Erk1/2 in starved mouse embryonic fibroblasts (MEFs) 45, 60, 75, and 90 min after infection with T3SS1^+^ or T3SS1^−^ V. parahaemolyticus. A pulse of p-Erk1/2 was observed early during infection with T3SS1^+^ but not T3SS1^−^ V. parahaemolyticus. (B) Immunoblot for total Egr1 in starved MEFs 45, 60, 75, and 90 min after infection with POR3:T3SS1^+^ or POR3:T3SS1^−^. A rise in Egr1 protein levels over time was observed only in T3SS1^+^-infected MEFs. (C) Immunoblot for p-Erk1/2 and total Erk1/2 in starved MEFs 45, 60, 75, and 90 min after infection with T3SS1^+^Δ*vopQ* or T3SS1^+^Δ*vopS* V. parahaemolyticus. T3SS1^+^Δ*vopQ* did not induce Erk1/2 phosphorylation while T3SS1^+^Δ*vopS* induced prolonged Erk1/2 phosphorylation. (D) Immunoblot for total Egr1 in starved MEFs 45, 60, 75, and 90 min after infection with V. parahaemolyticus T3SS1^+^Δ*vopQ* or T3SS1^+^Δ*vopS*. No rise in Egr1 protein levels was observed in T3SS1^+^Δ*vopQ*-infected MEFs, while T3SS1^+^Δ*vopS* caused an increase in Egr1 protein levels. (E) Immunoblot showing p-Erk1/2 and total Erk1/2 in starved MEFs infected with T3SS1^+^, T3SS1^+^*vopQ*^S200P^, T3SS1^+^Δ*vopQ*, and T3SS1^+^Δ*vopQ*+p*vopQ* V. parahaemolyticus strains for 45, 60, 75, and 90 min. No pulse of pERK1/2 was observed in T3SS1^+^*vopQ*^S200P^-infected MEFs. (F) Immunoblot for total Egr1 in starved MEFs infected for 45, 60, 75, and 90 min with the same V. parahaemolyticus strains as in panel E. T3SS1^+^*vopQ*^S200P^ did not trigger an increase Egr1 protein levels in infected MEFs. In panels B, D, and F, the target band is marked with a red line, and background bands are indicated with a blue star. Blots are representative of 3 independent experiments.

To test if VopQ induces *EGR1* and *FOS* expression by activating the ERK1/2 MAPK pathway early during infection, we starved MEFs to remove basal ERK1/2 phosphorylation, infected them with V. parahaemolyticus T3SS1^+^ or V. parahaemolyticus T3SS1^−^ for 45, 60, 75, and 90 min, and probed for phospho-ERK1/2 as well as for the presence of downstream Egr1 by Western blotting. We found that the T3SS1 induced a pulse of ERK1/2 phosphorylation that peaked around 45 min and had completely disappeared by 90 min postinfection (Fig. 2A). Total Egr1 protein levels began to rise 60 min postinfection and reached their maximum at 90 min postinfection, which is an expected pattern of expression when the time for transcription and translation of the induced *Egr1* gene is taken into account (Fig. 2B). Using the MEK1/2 inhibitor U0126, we found that T3SS1-induced *Egr1* expression was dependent on MEK1/2 activity in MEFs, similar to what was previously reported for PHDFs (Fig. S3B) (18).

We repeated the infection time course with the V. parahaemolyticus T3SS1^+^Δ*vopQ* strain and found that the T3SS1-induced pulse of ERK1/2 phosphorylation was indeed dependent on VopQ, as was the increase in total Egr1 protein levels (Fig. 2C and D). When MEFs were infected with T3SS1^+^Δ*vopS*, we observed not only an amplified induction of ERK1/2 phosphorylation and Egr1 production but also an extended duration of ERK1/2 phosphorylation (Fig. 2C and D). Complementing T3SS1^+^Δ*vopS* with a wild-type copy of *vopS* reverted the induced ERK1/2 phosphorylation pattern to that observed during T3SS1^+^ infection (Fig. S3C). Interestingly, complementation of T3SS1^+^Δ*vopS* with the catalytically dead *vopS*^H348A^ allele did not result in a reversion of the included ERK1/2 phosphorylation pattern (Fig. S3C). This observation is consistent with previous work that found that VopS’s AMPylation of Rho GTPases inhibits host ERK1/2 and JNK MAPK pathways (17). This early pulse of VopQ-dependent ERK1/2 MAPK signaling in MEFs is distinct from the previously reported VopQ-dependent ERK1/2 phosphorylation in Caco-2 cells at late infection time points, 3 to 4 h postinfection, as those assays were performed with V. parahaemolyticus strains that encoded functional T3SS1, T3SS2, and TdhAS hemolysins (21). Taken together, these data support the model that the combined actions of VopQ and VopS create a pulse of ERK1/2 MAPK signaling that is restricted to early infection time points, resulting in the controlled expression of downstream transcription factors EGR1 and FOS.

If VopQ and VopS work together to fine-tune the host response, their co-occurrence in *Vibrio* genomes containing the T3SS1 gene cluster would be predicted to be high. To test this, we used the SyntTax server to identify all *Vibrio* strains that retained synteny in the T3SS1 gene neighborhood (Table S3). We identified 58 *Vibrio* strains representing 8 species containing the T3SS1 gene cluster and found that 91.4% of genomes containing *vopQ* also contained *vopS* (53/58) (Table S3). We found that these genes co-occur in diverse *Vibrio* species, including V. parahaemolyticus, Vibrio diabolicus, Vibrio antiquarius, Vibrio campbellii, and Vibrio alginolyticus (Fig. S4). Interestingly, the 5 genomes containing *vopQ* but lacking *vopS* belonged to two *Vibrio* species, Vibrio harveyi and Vibrio tubiashii.

10.1128/mSystems.00872-20.4 FIG S4 Co-occurrence of *vopQ* and *vopS* in *Vibrio* strains with T3SS1 synteny. Depiction of the T3SS1 gene neighborhood in representative *Vibrio* strains identified to retain synteny in this region by the SyntTax web server. Genes homologous to VopQ (blue) and VopS (brown) are outlined. Only two species (5 strains) possess VopQ without VopS, Vibrio tubiashii (*V. tubiashii* ATCC 19109 [aa7721051 C1]) and Vibrio harveyi (4 strains with identical T3SS1 gene neighborhoods: V. harveyi 345 [aa28502951 C1], V. harveyi ATCC 33843 [392 MAV aa7701152 C1], V. harveyi ATCC 43516 [aa15584352 C1], and V. harveyi QT520 [aa19084352 C1]). Download FIG S4, TIF file, 1.1 MB. Copyright © 2021 De Nisco et al. 2021 De Nisco et al. This content is distributed under the terms of the Creative Commons Attribution 4.0 International license.

10.1128/mSystems.00872-20.10 TABLE S3 SyntTax VopQ and VopS co-occurrence in *Vibrio* genomes with T3SS1 synteny. Download Table S3, XLSX file, 0.1 MB. Copyright © 2021 De Nisco et al. 2021 De Nisco et al. This content is distributed under the terms of the Creative Commons Attribution 4.0 International license.

### VopQ-induced prosurvival signaling is independent of endosomal deacidification.

Next, we wanted to understand how VopQ could activate ERK1/2 MAPK signaling in the host. The VopQ channel deacidifies vacuolar and lysosomal compartments but also inhibits homotypic fusion of yeast vacuoles, a model for Rab GTPase- and SNARE-dependent fusion between the lysosome and autophagosome (8, 22). Mutation of serine 200 to a proline creates a mutant, VopQ^S200P^, that is still able to neutralize the vacuole or lysosome but can no longer block fusion (8). This observation is likely due to reduced binding of VopQ^S200P^ to the V-ATPase (19). To test if VopQ’s activation of ERK1/2 MAPK signaling was caused by one or both of these functions, we exchanged the chromosomal copy of the *vopQ* gene in the V. parahaemolyticus T3SS1^+^ strain with a version encoding VopQ^S200P^, creating V. parahaemolyticus strain T3SS1^+^*vopQ*^S200P^. We tested the cytotoxicity of this strain during MEF infection and found that the VopQ^S200P^ mutant was no less lethal than wild-type VopQ (Fig. S3A). However, unlike its parent strain, V. parahaemolyticus T3SS1^+^*vopQ*^S200P^ was unable to induce ERK1/2 phosphorylation and downstream production of Egr1 in MEFs (Fig. 2E and F). Notably, treatment of MEFs with chloroquine, a drug that prevents lysosomal acidification, was not able to induce phosphorylation of ERK1/2 (Fig. 2E). These data suggest that ERK1/2 MAPK signaling is not activated by lysosomal deacidification alone and is instead dependent on VopQ’s strong physical interaction with the V-ATPase. We therefore considered two models by which VopQ could induce ERK1/2 MAPK signaling. In the first, VopQ’s inhibition of lysosome-autophagosome fusion directly activates ERK1/2 MAPK signaling. In the second, VopQ manipulates another pathway upstream of both lysosome-autophagosome fusion and ERK1/2, thereby altering these two pathways in parallel.

### VopQ-induced prosurvival ERK1/2 signaling is dependent on IRE1.

**FIG 3:**
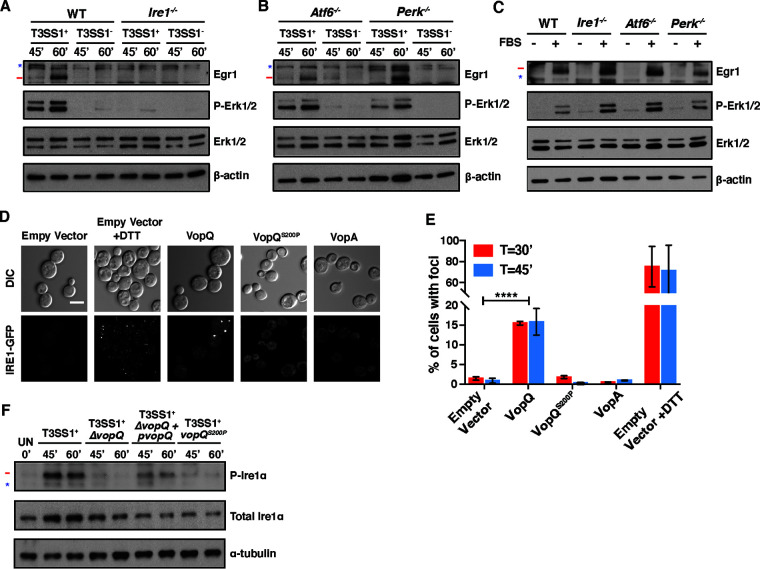
Activation of ERK1/2 MAPK signaling by VopQ is dependent on IRE1 activation. (A) Immunoblots for total Egr1, p-Erk1/2, and total Erk1/2 in starved wild-type (WT) and *Ire1*^−/−^ MEFs infected for 45 and 60 min with V. parahaemolyticus T3SS1^+^ or V. parahaemolyticus T3SS1^−^. Erk1/2 phosphorylation and increased Egr1 protein levels were not observed in *Ire1*^−/−^ MEFs. (B) Immunoblots for total Egr1, p-Erk1/2, and total Erk1/2 in starved *Atf6*^−/−^ and *Perk*^−/−^ MEFs infected for 45 and 60 min with V. parahaemolyticus T3SS1^+^ or V. parahaemolyticus T3SS1^−^. (C) Immunoblots for total Egr1, p-Erk1/2, and total Erk1/2 in starved (−) or FBS-stimulated (+) WT, *Ire1*^−/−^, *Atf6*^−/−^, and *Perk*^−/−^ MEFs. (D) Representative micrograph of Ire1p-GFP cluster formation in yeast after 45 min of DTT treatment or effector expression. Bar, 5 μm. (E) Quantification of the data in panel D showing the average percentage of cells (*n *= 100) with Ire1p-GFP foci from 3 independent experiments. Error bars represent standard errors of the means (SEM). *P* values were calculated by an unpaired *t* test (****, *P < *0.0001). (F) Immunoblot for p-Ire1α and total Ire1α in MEFs infected with T3SS1^+^, T3SS1^+^Δ*vopQ*, T3SS1^+^Δ*vopQ*+p*vopQ*, and T3SS1^+^*vopQ*^S200P^ V. parahaemolyticus strains for 45 and 60 min. In panels A to C and F, the target band is marked with a red line, and background bands are indicated with a blue star. Blots are representative of 3 independent experiments.

To test these models, we aimed to identify a signaling pathway that could be upstream of both autophagosome-lysosome fusion and ERK1/2 MAPK signaling. The unfolded protein response was previously linked to both of these processes through IRE1’s connections to the UPR and the ER-associated protein degradation (ERAD) pathway (23–26). Moreover, induction of ER stress with a proline analogue was previously shown to partially stimulate IRE1-dependent ERK1/2 activation to promote cell survival by an undefined mechanism (27). To examine if the UPR played a role, we tested if VopQ’s activation of ERK1/2 and downstream EGR1 production was dependent on any of the three branches of UPR: IRE1, activating transcription factor 6 (ATF6), or PKR-like ER kinase (PERK) (28). We infected *IRE1*^−/−^ MEFs, *Atf6*^−/−^ MEFs, *PERK*^−/−^ MEFs, and wild-type MEFs with V. parahaemolyticus T3SS1^+^ and V. parahaemolyticus T3SS1^−^ and monitored phosphorylation of ERK1/2 and accumulation of Egr1 by Western blotting. While infection of *Atf6*^−/−^ MEFs, *PERK*^−/−^ MEFs, and wild-type MEFs with V. parahaemolyticus T3SS1^+^ resulted in robust ERK1/2 phosphorylation 45 and 60 min postinfection, phosphorylated ERK1/2 was not detected in *IRE1*^−/−^ MEFs infected with V. parahaemolyticus T3SS1^+^ (Fig. 3A and B). Consistent with this observation, accumulation of Egr1 was also not observed in *IRE1*^−/−^ MEFs infected with V. parahaemolyticus T3SS1^+^ (Fig. 3A). VopQ was still cytotoxic to *IRE1*^−/−^ MEFs (Fig. S5A), consistent with the maintained cytotoxicity of VopQ^S200P^ in wild-type MEFs despite its inability to activate ERK1/2 (Fig. S3A). These data suggest that VopQ induces ERK1/2 phosphorylation by an IRE1-dependent mechanism.

10.1128/mSystems.00872-20.5 FIG S5 VopQ-mediated cytotoxicity is conserved in UPR mutant cell lines, and VopQ but not VopQ^S200P^ inhibits growth of BY471 IRE1-GFP yeast. Calculation of cytotoxicity by quantification of lactate dehydrogenase (LDH) release by WT or *Ire1*^−/−^ (a), *Atf6*^−/−^ (b), or *Perk*^−/−^ (c) MEFs infected with V. parahaemolyticus T3SS1^+^ or T3SS1^+^Δ*vopQ*. The percent cytotoxicity was calculated relative to uninfected (negative) and Triton X-100-treated (positive) controls. Error bars show SD, and data are means for 9 replicates (3 biological replicates and 3 technical replicates). *P* values were calculated by one-way ANOVA and Dunnett’s multiple-comparison test (****, *P < *0.0001; n.s., not significant). (d) Serial dilutions of yeast strain BY4741 IRE1-GFP harboring pRS416-Gal1-FLAG, pRS416-Gal1-FLAG-VopQ, pRS416-Gal1-FLAG-VopQ^S200P^, or pRS416-Gal1-VopA-FLAG in CSM medium lacking histidine and uracil, supplemented with either 2% glucose or 2% galactose. Download FIG S5, TIF file, 1.7 MB. Copyright © 2021 De Nisco et al. 2021 De Nisco et al. This content is distributed under the terms of the Creative Commons Attribution 4.0 International license.

VopQ-mediated cytotoxicity was also conserved in the *Atf6*^−/−^ and *PERK*^−/−^ MEFs (Fig. S5B and C). Both the *Atf6*^−/−^ and *PERK*^−/−^ cell lines exhibited the same pattern of ERK1/2 phosphorylation and Egr1 accumulation upon T3SS1^+^ infection as wild-type MEFs (Fig. 3B), indicating that VopQ’s activation of ERK1/2 was specifically IRE1 dependent. Finally, we stimulated wild-type, *IRE1*^−/−^, *Atf6*^−/−^, and *PERK*^−/−^ MEFs with fetal bovine serum (FBS) to assess whether the well-described growth factor-stimulated ERK1/2 MAPK signaling was functional in these cell lines (29). FBS-stimulated ERK1/2 phosphorylation and downstream Egr1 expression were observed in all cell lines (Fig. 3C). These data strongly support our model that VopQ’s IRE1-dependent activation of ERK1/2 occurs through a pathway that is separate from the established growth factor-stimulated pathway mediated by Ras and Raf (29).

### VopQ expression results in IRE1 activation in yeast.

Next, we asked if VopQ activates IRE1. VopQ toxicity is dependent on the assembly c subunit of the V_o_ V-ATPase in yeast independent of the vacuolar localization of the complex. This led us to hypothesize that interaction of VopQ and the c subunit ring could also take place in the ER, where the V_o_ complex initially forms (19). In addition, the ER is the source of membranes for autophagosomes; thus, a block in autophagic flux may also perturb the protein-to-lipid ratio of the ER (30). We predicted that if either of these scenarios occurred, the membrane perturbations caused by VopQ might activate the UPR, which is mediated by IRE1 in yeast, either through the disruption of the ER lumen environment or through the activation of lipid bilayer stress (31–34). IRE1 is a type I ER-resident transmembrane protein that contains a protein kinase and an endoribonuclease domain in its cytoplasmic region (35). IRE1 also contains an amphipathic helix which can sense perturbations in the lipid bilayer, leading to activation and initiation of the UPR (32).

To test this hypothesis, we assessed the clustering of IRE1 in yeast by visualizing endogenously expressed IRE1p-GFP (9). Plasmids carrying galactose-inducible genes encoding wild-type VopQ, the V-ATPase binding mutant VopQ^S200P^, and VopA were transformed into the BY4741 IRE1-green fluorescent protein (GFP) yeast strain. VopA is a V. parahaemolyticus T3SS2 effector that kills yeast by a mechanism that is distinct from VopQ and was included as a control (36). Upon galactose induction, serial growth assays showed that VopQ and VopA both inhibited growth in the BY4741 IRE1-GFP strain while VopQ^S200P^ and vector alone control did not (Fig. S5D). We then monitored IRE1p-GFP clustering, or focus formation, at 30 and 45 min after galactose induction by confocal microscopy. Treatment with dithiothreitol (DTT) for the same period was used as a positive control for UPR stress. Yeast expressing VopQ but not VopQ^S200P^ or VopA induced IRE1p-GFP focus formation (Fig. 3D). IRE1p-GFP foci formation was observed, on average, in about 70% of DTT-treated cells compared to about 15% in cells expressing VopQ (Fig. 3E). This difference was expected, because DTT treatment is homogeneous, whereas expression of VopQ is stochastic (37). Our data indicate that expression of VopQ in yeast results in IRE1 activation.

### VopQ causes induction of the IRE1 branch of the UPR of mammalian cells during infection.

We next asked if VopQ also activates IRE1 in mammalian cells during infection. IRE1 is normally sequestered by BiP but is released upon UPR activation, when it oligomerizes, *trans*-autophosphorylates, and activates its endoribonuclease activity, resulting in the nonconventional splicing of X-box binding protein 1 (XBP1) mRNA in mammalian cells (38–40). To determine if the IRE1 branch of the UPR was activated by VopQ at early infection time points, we measured levels of phospho-IRE1α in MEFs by Western blotting at 45 and 60 min postinfection. Our results indicate that V. parahaemolyticus T3SS1^+^ induces IRE1α phosphorylation in a VopQ-dependent manner (Fig. 3F) and that T3SS1^+^*vopQ*^S200P^ is not able to induce IRE1α phosphorylation. Taken together, these data indicate that the V-ATPase binding activity of VopQ activates the IRE1 branch of the UPR in both MEFs and yeast.

### VopQ-induced prosurvival ERK signaling is dependent on IRE1 kinase activity.

**FIG 4:**
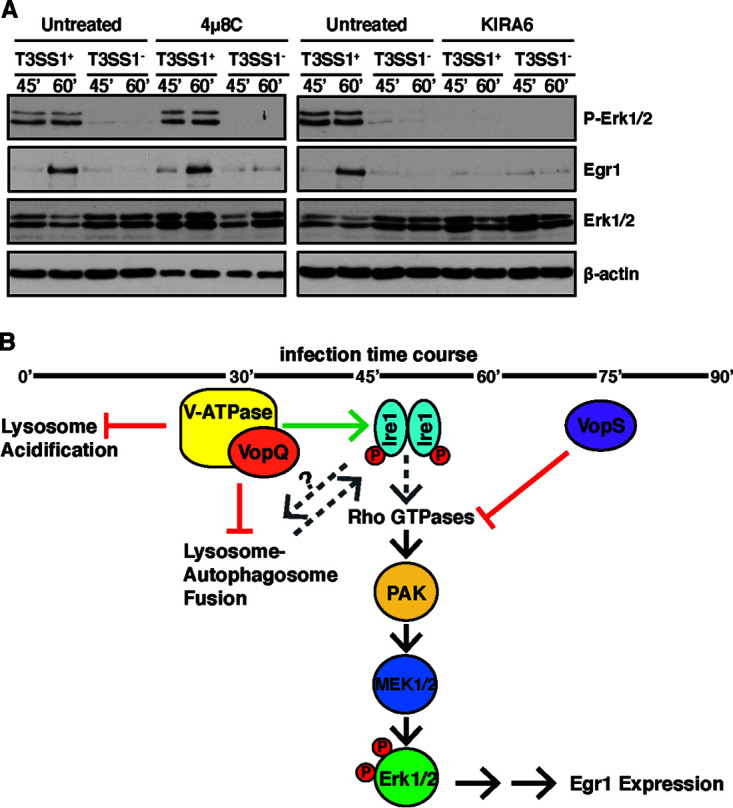
Activation of ERK1/2 MAPK signaling by VopQ is dependent on IRE1 kinase activity. (A) Immunoblots for total Egr1, p-Erk1/2, and total Erk1/2 in starved wild-type (WT) MEFs that were untreated or treated with 4μ8c or KIRA6 inhibitors for 24 h and 1 h, respectively, and infected for 45 and 60 min with V. parahaemolyticus T3SS1^+^ or T3SS1^−^. Erk1/2 phosphorylation and increased Egr1 protein levels are not observed in MEFs treated with KIRA6 kinase inhibitor. Blots are representative of 3 independent experiments. (B) Model for IRE1-dependent modulation of Erk1/2 MAPK signaling by VopQ and VopS during V. parahaemolyticus infection. The green arrow indicates activation, and red lines depict inhibition. Dashed lines indicate postulated connections in the model requiring future study. Specifically, it is possible that Rho GTPases may be upstream of Ire1, and how VopQ’s inhibition of autophagic flux affects Ire1 signaling and vice versa is unclear.

Next, we wanted to further determine if the catalytic activities of IRE1 are required for VopQ-induced ERK1/2 signaling. IRE1 contains both protein kinase and an endoribonuclease domain in its cytoplasmic region (35). To test if the kinase or endonuclease activity of IRE1 is required for ERK signaling, MEFs were treated before V. parahaemolyticus infection with KIRA6 or 4μ8c, an IRE1-specific kinase inhibitor and a potent inhibitor of IRE1 RNase activity, respectively (Fig. 4A). Strikingly, ERK1/2 phosphorylation and Egr1 protein expression were not observed in infected MEFs treated with the kinase inhibitor KIRA6. However, 4μ8c treatment appeared to have no effect on VopQ-induced ERK1/2 phosphorylation and Egr1 protein expression. Taken together, these data indicate that the prosurvival ERK signaling induced by VopQ during V. parahaemolyticus infection is dependent on IRE1 kinase activity.

## DISCUSSION

By studying the function of two effectors of the T3SS1 of the seafood-borne pathogen V. parahaemolyticus, we have shown that T3SS effectors act together to systematically manipulate the host response. We observe that the effector VopQ is responsible for the T3SS1-mediated activation of cell survival and repression of cell death networks and another effector, VopS, is responsible for dampening this response. Of note, VopS has been determined to AMPylate and thereby inactivate Rac, a known activator of MEK1/2 mediated ERK signaling (9). Furthermore, in Vibrio alginolyticus, a *Vibrio* species closely related to V. parahaemolyticus, VopS was found to be required for the rapid induction of apoptosis in infected cells (41). The association of VopQ with the V-ATPase elicits early activation of ERK1/2 phosphorylation that is turned off by the delayed temporal action of VopS (Fig. 4B).

Manipulation of the V-ATPase to affect signaling in the cell was previously observed with oncogenic Ras, which induces a Rac-dependent plasma membrane ruffling and micropinocytosis. However, in the case of oncogenic Ras, activation of Rac is dependent on the activity of the V-ATPase along with its relocation to the plasma membrane (42). Importantly, the activation ERK1/2 MAPK signaling by VopQ does not occur through the growth factor-inducible Ras-mediated pathway but instead is dependent on the kinase activity of the ER stress sensor and cell fate executor IRE1 (Fig. 4B). Activation of ERK1/2 MAPK signaling specifically through the IRE1 branch of the UPR by a bacterial effector has not previously been reported.

The evolutionary conservation of *vopQ* and *vopS* in *Vibrio* species retaining synteny in the T3SS1 neighborhood suggests that their concerted function is important for proper modulation of the host response. Effector pairs with opposing effects yet synergistic effects during infection are not unknown. YopJ and YopM of Yersinia pestis were previously described as having opposing effects on interleukin signaling and caspase-1 processing which synergistically suppressed proinflammatory cytokines during infection (43). In *Salmonella*, the effectors SptP and SopE act as respective GTP-activating proteins (GAPs) and guanine nucleotide exchange factors (GEFs) for Cdc42 and Rac. Rapid degradation of SopE allows the temporal regulation of Cdc42 and Rac activity during *Salmonella* infection (44). Furthermore, Legionella pneumophila utilizes several such pairs of effectors, such as SidM/DrrA and LepB, SidH and LubX, and AnkX and Lem3 (45–51). Together, these effector pairs coordinate the establishment, maintenance, and properly timed escape from the legionella-containing vacuole (LCV), the environmental niche *Legionella* requires to replicate (52, 53).

The targeting of autophagy, UPR, and MAPK signaling together is not unprecedented for pathogens. For example, the mycotoxin patulin was found to manipulate these pathways though the inhibition of cathepsin B and cathepsin D, which leads to an accumulation of p62. Increased p62-mediated autophagy activates the PERK and IRE1 branches of the UPR through increased reactive oxygen species production. UPR activation then results in activation of ERK1/2 and phosphorylation of BAD, resulting in increased survival of host cells (54). Several viruses rely on the interplay of UPR and MAPK signaling for virulence as well. Dengue virus (DENV) relies on UPR activation of JNK signaling to induce autophagy and increase viral load of infected cells. Treatment with a JNK inhibitor decreased viral titers and reduced symptoms of DENV2 in mice (55). Recently the coronavirus infectious bronchitis virus (IBV) was found to rely on the activation of IRE1 and ERK1/2, but not XBP1 or JNK, for the induction of autophagic flux and prosurvival signaling during IBV infection (56).

We and others have observed that the ERK1/2 MAPK signaling can be regulated either directly or indirectly through the IRE1 branch of the UPR (54). However, it remains unclear if VopQ’s induction of IRE1 signaling is caused by the manipulation of autophagy or via localized perturbations in the ER membrane caused by VopQ’s interaction with assembly intermediates of the V-ATPase V_o_ subcomplex in the ER. Deciphering the epistatic relationship between VopQ’s association with the V-ATPase and activation of IRE1 will be the subject of future studies that will be important for understanding the role of the V-ATPase–UPR–MAPK feedback network in both cellular homeostasis and bacterial infection. Similarly, the interaction of VopQ with the V-ATPase at the ER should be further studied to determine if this interaction is sufficient to activate IRE1’s lipid bilayer stress response and ERK1/2 MAPK signaling independently of autophagy (33, 57). Other groups have hypothesized that Ire1’s interaction with the adapter protein Nck plays an important role in activation of ERK1/2 signaling upon ER stress; however, whether IRE1’s kinase or endonuclease activity is needed was unknown (27). Our studies indicate that the kinase activity of IRE1 is required, but the mechanism by which IRE1 kinase activity leads to ERK1/2 MAPK signaling is still poorly understood. Future experiments to dissect the role of Nck and Rho GTPase activation in this process would be a valuable addition to understanding this molecular mechanism of IRE1-induced, growth factor-independent ERK1/2 MAPK signaling.

## MATERIALS AND METHODS

### Bacterial strains and culture conditions.

The Vibrio parahaemolyticus POR3 (POR1Δ*vcrD2*) and POR4 (POR1Δ*vcrD1*/*vcrD2*) strains were generously provided by Tetsuya Iida and Takeshi Honda of Osaka University. *Vibrio* strains were cultured at 30°C in MLB (Luria-Bertani broth with 3% NaCl). All V. parahaemolyticus strains except V. parahaemolyticus T3SS1^+^*vopQ*^S200P^ were from previous studies (Table S1). The T3SS1^+^*vopQ*^S200P^ strain was created by cloning the *vopQ*^S200P^ allele (8) flanked by the nucleotide sequences 1 kb upstream and 1 kb downstream of *vopQ* (vp1680) into pDM4, a Cm^r^ OriR6K suicide plasmid. Escherichia coli S17 (λ*pir*) was used to conjugate the resulting plasmid into the POR3 strain, and transconjugants were selected on medium containing 25 μg/ml chloramphenicol. Bacteria were then counterselected on 15% sucrose, and insertion of the *vopQ^S200P^* allele was confirmed by PCR.

### Mammalian cell culture.

Primary adult dermal fibroblasts (PHDFs; ATCC PCS-201-01) were purchased from ATCC and revived and maintained at 5% CO_2_ and 37°C in low-serum primary fibroblast medium (ATCC) according to ATCC instructions. Wild-type mouse embryonic fibroblasts (MEFs) were a gift from Jenna Jewell, and *IRE1*^−/−^, *PERK*^−/−^ and *Atf6*^−/−^ MEFs were kindly provided by Fumiko Urano. MEFs were maintained at 5% CO_2_ and 37°C in high-glucose Dulbecco’s modified Eagle’s medium (DMEM; Gibco) supplemented with 10% (vol/vol) fetal bovine serum (Sigma-Aldrich), 1% (vol/vol) penicillin-streptomycin-glutamine, and 1% (vol/vol) sodium pyruvate.

### Yeast strains and plasmids.

All yeast genetic techniques were performed by standard procedures described previously (58). All strains were cultured in either rich (1% yeast extract, 2% peptone, and 2% dextrose [YPD]) or complete synthetic minimal (CSM) medium (Sigma) lacking appropriate amino acids with 2% dextrose, 2% raffinose, or 2% galactose. Yeasts were serially diluted and spotted onto agar plates to assay fitness and temperature sensitivity per standard techniques. Yeast strains used in this study were BY4741 (MAT**a** *his3Δ0 leu2Δ0 met15Δ0 ura3Δ0*) and BY4741 IRE1-GFP (MAT**a** *his3Δ0 leu2Δ0 met15Δ0 ura3Δ0 IRE1-GFP*) (Thermo Fisher) as indicated.

Plasmids pRS416-Gal1-FLAG-VopQ and pRS416-Gal1-FLAG-VopQ^S200P^ were generated by subcloning pRS413-Gal1-VopQ and pRS413-Gal1-VopQ^S200P^ (8) into the BamHI and EcoRV sites of pRS416-Gal1. Plasmid pRS416-Gal1-VopA-FLAG was generated by subcloning pRS413-Gal1-VopA-FLAG (36) into the EcoRI and XhoI sites of pRS416-Gal1.

### Infection of PHDFs for RNA sequencing and quantitative RT-PCR.

For RNA sequencing, PHDFs were seeded onto 6-well plates at a density of 1 × 10^5^ cells/ml and grown for 18 to 20 h to ∼80% confluence. Overnight V. parahaemolyticus cultures were normalized to an optical density at 600 nm (OD_600_) of 0.2 and subcultured to an OD_600_ of 0.6. Bacteria were pelleted, resuspended in unsupplemented DMEM, and grown at 37°C for 45 min to preinduce T3SS1 expression (16). PHDFs were washed with unsupplemented DMEM and then infected with preinduced V. parahaemolyticus strains at a multiplicity of infection (MOI) of 10. Plates were centrifuged at 1,000 × *g* for 5 min to synchronize infection and incubated at 37°C and 5% CO_2_. At 90 min postinfection, RNAprotect cell reagent (Qiagen) was added to stop the infection and preserve the RNA. Cells were harvested by scraping and pooled, and pellets were resuspended in RLT-plus buffer (Qiagen) and stored at −80°C. The same infection protocol was followed for quantitative PCR (qPCR) experiments with PHDFs and MEFs. For MEK1/2 inhibition qPCR experiments, MEFs were incubated with 10 μM U0126 (Cell Signaling) or 10 μM dimethyl sulfoxide (DMSO) (vehicle) during and for 1 h prior to infection.

### RNA isolation and RNA sequencing.

RNA isolation was performed as for PHDFs and MEFs. Cells were lysed with 27-gauge, 1/2-in. needles and then homogenized with QIAshredder columns (Qiagen). Total RNA from triplicate experiments was purified with the RNeasy Plus kit (Qiagen). The quality of purified total RNA samples was determined with an Agilent 2100 Bioanalyzer, and only samples with an RNA integrity number (RIN) of 9 or higher were used. RNA concentration was measured with a Qubit fluorimeter prior to library prep. Four micrograms of total DNase-treated RNA was run through the TruSeq Stranded Total RNA LT sample prep kit from Illumina as previously described (18). Samples were quantified by Qubit before being normalized, pooled, and then sequenced on the Illumina HiSeq 2500 sequencer with SBS v3 reagents. Each sample was sequenced at a depth of at least 25 million 50-nucleotide single-end reads.

### Infection of MEFs for immunoblotting.

For ERK1/2 and Egr1 immunoblotting experiments, MEFs were starved in unsupplemented DMEM for 1 h prior to infection/treatment to remove background growth factor-stimulated MAPK signaling. MEFs were not starved for in phospho-IRE1α experiments in order to prevent background activation of the UPR by nutritional stress. Cells were then infected at an MOI of 10 with V. parahaemolyticus as described above or with treated DMEM supplemented with 10% (vol/vol) fetal bovine serum (FBS; Sigma-Aldrich). To inhibit IRE1 nuclease and kinase activities, cells were treated with 100 μM 4μ8c for 24 h and 100 μM KIRA6 for 1 h before infection, respectively.

### Immunoblotting.

MEFs were infected as described above, washed with 1× ice-cold phosphate-buffered saline (PBS), and collected by scraping at each time point. Collected cells were pelleted (1,000 × *g*), washed twice in ice-cold 1× PBS, and lysed in radioimmunoprecipitation assay (RIPA) buffer (50 mM Tris [pH 8.0], 150 mM NaCl, 5 mM EDTA, 1% Nonidet P-40, 0.5% sodium deoxycholate, 0.1% SDS) with protease and phosphatase inhibitors (Roche Applied Science) for 20 min on ice. Total protein concentration of lysed supernatants was determined by the Bradford assay, and all samples were normalized for total protein prior to gel electrophoresis and immunoblotting. Total ERK1/2 and phospho-ERK1/2 were detected with Cell Signaling Technologies (CST) p44/42 MAPK (137F5) and P-p44/42 MAPK T202/Y204 (197G2) primary antibodies, respectively. Early growth response 1 (EGR1) was detected using CST EGR1 antibody (15F7), and β-actin was detected by Sigma-Aldrich A2228 monoclonal anti-β-actin. Total IRE1α and phospho-IRE1α were detected by CST IRE1α (14C10) and Novus IRE1α (pSer724) antibodies, respectively. α-Tubulin was detected by Santa Cruz α-tubulin antibody (B-7). Secondary horseradish peroxidase (HRP)-conjugated antibodies used were donkey anti-rabbit immunoglobulin (GE Healthcare) and goat anti-mouse immunoglobulin (Sigma-Aldrich).

### IRE1p-GFP clustering assay.

Yeast strains were grown to mid-log phase (OD_600_ ∼ 0.5) in CSM medium lacking uracil with 2% raffinose. Cultures were then treated with 2% galactose, 2% raffinose, or 2% raffinose with 5 mM DTT for 30 or 45 min. Cultures were collected, resuspended in 1× PBS, and fixed with 4% paraformaldehyde. Confocal images were acquired using a Zeiss LSM800 with Zen software. Images were processed with ImageJ (National Institutes of Health) and Adobe Photoshop CS6. For quantification, the presence of IRE1-GFP foci was scored in 100 cells per experiment over three independent experiments.

### Statistical methods.

For RNA sequencing DE analysis, statistical cutoffs were as follows: false discovery rate (FDR), ≤0.01; log_2_ counts per million (log_2_CPM), ≥0; and absolute value of fold change (FC), ≥1.5. For IPA and biological network analysis, the *P* values are presented as −log(*P* value) and the cutoff for significance was a *P* value of <0.05. FDRs and *P* values are reported in Data Set S1, sheets 1 to 4. For quantitative RT-PCR, *P* values were calculated by one-way analysis of variance (ANOVA) and Dunnett’s multiple-comparison test. For the quantification of IRE1-GFP clustering, *P* values were calculated by Student’s unpaired *t* test (two tailed). Additional materials and methods are available in the supplemental material.

### Data availability.

Complete RNA-sequencing data have been deposited on the Gene Expression Omnibus server (GSE120273).

10.1128/mSystems.00872-20.6 TEXT S1 Supplemental materials and methods. Download Text S1, DOCX file, 0.03 MB. Copyright © 2021 De Nisco et al. 2021 De Nisco et al. This content is distributed under the terms of the Creative Commons Attribution 4.0 International license.

## References

[1] Alto NM, Orth K. 2012. Subversion of cell signaling by pathogens. Cold Spring Harb Perspect Biol 4:a006114. doi:10.1101/cshperspect.a006114. 22952390 PMC3428769

[2] Burdette DL, Yarbrough ML, Orvedahl A, Gilpin CJ, Orth K. 2008. Vibrio parahaemolyticus orchestrates a multifaceted host cell infection by induction of autophagy, cell rounding, and then cell lysis. Proc Natl Acad Sci U S A 105:12497–12502. doi:10.1073/pnas.0802773105. 18713860 PMC2527940

[3] Makino K, Oshima K, Kurokawa K, Yokoyama K, Uda T, Tagomori K, Iijima Y, Najima M, Nakano M, Yamashita A, Kubota Y, Kimura S, Yasunaga T, Honda T, Shinagawa H, Hattori M, Iida T. 2003. Genome sequence of Vibrio parahaemolyticus: a pathogenic mechanism distinct from that of V cholerae. Lancet 361:743–749. doi:10.1016/S0140-6736(03)12659-1. 12620739

[4] de Souza Santos M, Orth K. 2014. Intracellular Vibrio parahaemolyticus escapes the vacuole and establishes a replicative niche in the cytosol of epithelial cells. mBio 5:e01506-14. doi:10.1128/mBio.01506-14. 25205094 PMC4173779

[5] Park KS, Ono T, Rokuda M, Jang MH, Okada K, Iida T, Honda T. 2004. Functional characterization of two type III secretion systems of Vibrio parahaemolyticus. Infect Immun 72:6659–6665. doi:10.1128/IAI.72.11.6659-6665.2004. 15501799 PMC523034

[6] O'Boyle N, Boyd A. 2014. Manipulation of intestinal epithelial cell function by the cell contact-dependent type III secretion systems of Vibrio parahaemolyticus. Front Cell Infect Microbiol 3:114. 24455490 10.3389/fcimb.2013.00114 PMC3887276

[7] Ono T, Park KS, Ueta M, Iida T, Honda T. 2006. Identification of proteins secreted via Vibrio parahaemolyticus type III secretion system 1. Infect Immun 74:1032–1042. doi:10.1128/IAI.74.2.1032-1042.2006. 16428750 PMC1360304

[8] Sreelatha A, Bennett TL, Carpinone EM, O'Brien KM, Jordan KD, Burdette DL, Orth K, Starai VJ. 2015. Vibrio effector protein VopQ inhibits fusion of V-ATPase-containing membranes. Proc Natl Acad Sci U S A 112:100–105. doi:10.1073/pnas.1413764111. 25453092 PMC4291640

[9] Yarbrough ML, Li Y, Kinch LN, Grishin NV, Ball HL, Orth K. 2009. AMPylation of Rho GTPases by Vibrio VopS disrupts effector binding and downstream signaling. Science 323:269–272. doi:10.1126/science.1166382. 19039103

[10] Salomon D, Guo Y, Kinch LN, Grishin NV, Gardner KH, Orth K. 2013. Effectors of animal and plant pathogens use a common domain to bind host phosphoinositides. Nat Commun 4:2973. doi:10.1038/ncomms3973. 24346350 PMC4981085

[11] Sreelatha A, Orth K, Starai VJ. 2013. The pore-forming bacterial effector, VopQ, halts autophagic turnover. Autophagy 9:2169–2170. doi:10.4161/auto.26449. 24145145 PMC4028346

[12] Burdette DL, Seemann J, Orth K. 2009. Vibrio VopQ induces PI3-kinase-independent autophagy and antagonizes phagocytosis. Mol Microbiol 73:639–649. doi:10.1111/j.1365-2958.2009.06798.x. 19627496 PMC2733864

[13] Higa N, Toma C, Koizumi Y, Nakasone N, Nohara T, Masumoto J, Kodama T, Iida T, Suzuki T. 2013. Vibrio parahaemolyticus effector proteins suppress inflammasome activation by interfering with host autophagy signaling. PLoS Pathog 9:e1003142. doi:10.1371/journal.ppat.1003142. 23357873 PMC3554609

[14] Zhou X, Konkel ME, Call DR. 2010. Vp1659 is a Vibrio parahaemolyticus type III secretion system 1 protein that contributes to translocation of effector proteins needed to induce cytolysis, autophagy, and disruption of actin structure in HeLa cells. J Bacteriol 192:3491–3502. doi:10.1128/JB.01493-09. 20418402 PMC2897656

[15] Nguyen AQ, Shimohata T, Hatayama S, Tentaku A, Kido J, Bui TMH, Uebanso T, Mawatari K, Takahashi A. 2020. Type III secretion effector VopQ of Vibrio parahaemolyticus modulates central carbon metabolism in epithelial cells. mSphere 5:e00960-19. doi:10.1128/mSphere.00960-19. 32188755 PMC7082145

[16] Broberg CA, Zhang L, Gonzalez H, Laskowski-Arce MA, Orth K. 2010. A Vibrio effector protein is an inositol phosphatase and disrupts host cell membrane integrity. Science 329:1660–1662. doi:10.1126/science.1192850. 20724587

[17] Woolery AR, Yu X, LaBaer J, Orth K. 2014. AMPylation of Rho GTPases subverts multiple host signaling processes. J Biol Chem 289:32977–32988. doi:10.1074/jbc.M114.601310. 25301945 PMC4239643

[18] De Nisco NJ, Kanchwala M, Li P, Fernandez J, Xing C, Orth K. 2017. The cytotoxic type 3 secretion system 1 of Vibrio rewires host gene expression to subvert cell death and activate cell survival pathways. Sci Signal 10:eaa14501. doi:10.1126/scisignal.aal4501. PMC5662193 28512145

[19] Peng W, Casey AK, Fernandez J, Carpinone EM, Servage KA, Chen Z, Li Y, Tomchick DR, Starai VJ, Orth K. 2020. A distinct inhibitory mechanism of the V-ATPase by Vibrio VopQ revealed by cryo-EM. Nat Struct Mol Biol 27:589–597. doi:10.1038/s41594-020-0429-1. 32424347

[20] Hodge C, Liao J, Stofega M, Guan K, Carter-Su C, Schwartz J. 1998. Growth hormone stimulates phosphorylation and activation of elk-1 and expression of c-fos, egr-1, and junB through activation of extracellular signal-regulated kinases 1 and 2. J Biol Chem 273:31327–31336. doi:10.1074/jbc.273.47.31327. 9813041

[21] Shimohata T, Nakano M, Lian X, Shigeyama T, Iba H, Hamamoto A, Yoshida M, Harada N, Yamamoto H, Yamato M, Mawatari K, Tamaki T, Nakaya Y, Takahashi A. 2011. Vibrio parahaemolyticus infection induces modulation of IL-8 secretion through dual pathway via VP1680 in Caco-2 cells. J Infect Dis 203:537–544. doi:10.1093/infdis/jiq070. 21177635 PMC3071237

[22] Nakamura S, Yoshimori T. 2017. New insights into autophagosome-lysosome fusion. J Cell Sci 130:1209–1216. doi:10.1242/jcs.196352. 28302910

[23] Yan MM, Ni JD, Song D, Ding M, Huang J. 2015. Interplay between unfolded protein response and autophagy promotes tumor drug resistance. Oncol Lett 10:1959–1969. doi:10.3892/ol.2015.3508. 26622781 PMC4579870

[24] Rashid HO, Yadav RK, Kim HR, Chae HJ. 2015. ER stress: autophagy induction, inhibition and selection. Autophagy 11:1956–1977. doi:10.1080/15548627.2015.1091141. 26389781 PMC4824587

[25] Darling NJ, Cook SJ. 2014. The role of MAPK signalling pathways in the response to endoplasmic reticulum stress. Biochim Biophys Acta 1843:2150–2163. doi:10.1016/j.bbamcr.2014.01.009. 24440275

[26] Cheng X, Liu H, Jiang CC, Fang L, Chen C, Zhang XD, Jiang ZW. 2014. Connecting endoplasmic reticulum stress to autophagy through IRE1/JNK/beclin-1 in breast cancer cells. Int J Mol Med 34:772–781. doi:10.3892/ijmm.2014.1822. 24970676

[27] Nguyen DT, Kebache S, Fazel A, Wong HN, Jenna S, Emadali A, Lee EH, Bergeron JJ, Kaufman RJ, Larose L, Chevet E. 2004. Nck-dependent activation of extracellular signal-regulated kinase-1 and regulation of cell survival during endoplasmic reticulum stress. Mol Biol Cell 15:4248–4260. doi:10.1091/mbc.e03-11-0851. 15201339 PMC515356

[28] Schroder M, Kaufman RJ. 2005. The mammalian unfolded protein response. Annu Rev Biochem 74:739–789. doi:10.1146/annurev.biochem.73.011303.074134. 15952902

[29] Katz M, Amit I, Yarden Y. 2007. Regulation of MAPKs by growth factors and receptor tyrosine kinases. Biochim Biophys Acta 1773:1161–1176. doi:10.1016/j.bbamcr.2007.01.002. 17306385 PMC2758354

[30] Davis S, Wang J, Ferro-Novick S. 2017. Crosstalk between the secretory and autophagy pathways regulates autophagosome formation. Dev Cell 41:23–32. doi:10.1016/j.devcel.2017.03.015. 28399396 PMC5493037

[31] Hou NS, Gutschmidt A, Choi DY, Pather K, Shi X, Watts JL, Hoppe T, Taubert S. 2014. Activation of the endoplasmic reticulum unfolded protein response by lipid disequilibrium without disturbed proteostasis in vivo. Proc Natl Acad Sci U S A 111:E2271–E2280. doi:10.1073/pnas.1318262111. 24843123 PMC4050548

[32] Halbleib K, Pesek K, Covino R, Hofbauer HF, Wunnicke D, Hanelt I, Hummer G, Ernst R. 2017. Activation of the unfolded protein response by lipid bilayer stress. Mol Cell 67:673–684.E8. doi:10.1016/j.molcel.2017.06.012. 28689662

[33] Volmer R, van der Ploeg K, Ron D. 2013. Membrane lipid saturation activates endoplasmic reticulum unfolded protein response transducers through their transmembrane domains. Proc Natl Acad Sci U S A 110:4628–4633. doi:10.1073/pnas.1217611110. 23487760 PMC3606975

[34] Covino R, Hummer G, Ernst R. 2018. Integrated functions of membrane property sensors and a hidden side of the unfolded protein response. Mol Cell 71:458–467. doi:10.1016/j.molcel.2018.07.019. 30075144

[35] Tirasophon W, Welihinda AA, Kaufman RJ. 1998. A stress response pathway from the endoplasmic reticulum to the nucleus requires a novel bifunctional protein kinase/endoribonuclease (Ire1p) in mammalian cells. Genes Dev 12:1812–1824. doi:10.1101/gad.12.12.1812. 9637683 PMC316900

[36] Trosky JE, Mukherjee S, Burdette DL, Roberts M, McCarter L, Siegel RM, Orth K. 2004. Inhibition of MAPK signaling pathways by VopA from Vibrio parahaemolyticus. J Biol Chem 279:51953–51957. doi:10.1074/jbc.M407001200. 15459200

[37] Raj A, van Oudenaarden A. 2008. Nature, nurture, or chance: stochastic gene expression and its consequences. Cell 135:216–226. doi:10.1016/j.cell.2008.09.050. 18957198 PMC3118044

[38] Kimata Y, Ishiwata-Kimata Y, Ito T, Hirata A, Suzuki T, Oikawa D, Takeuchi M, Kohno K. 2007. Two regulatory steps of ER-stress sensor Ire1 involving its cluster formation and interaction with unfolded proteins. J Cell Biol 179:75–86. doi:10.1083/jcb.200704166. 17923530 PMC2064738

[39] Bertolotti A, Zhang Y, Hendershot LM, Harding HP, Ron D. 2000. Dynamic interaction of BiP and ER stress transducers in the unfolded-protein response. Nat Cell Biol 2:326–332. doi:10.1038/35014014. 10854322

[40] Back SH, Schroder M, Lee K, Zhang K, Kaufman RJ. 2005. ER stress signaling by regulated splicing: IRE1/HAC1/XBP1. Methods 35:395–416. doi:10.1016/j.ymeth.2005.03.001. 15804613

[41] Zhao Z, Liu J, Deng Y, Huang W, Ren C, Call DR, Hu C. 2018. The Vibrio alginolyticus T3SS effectors, Val1686 and Val1680, induce cell rounding, apoptosis and lysis of fish epithelial cells. Virulence 9:318–330. doi:10.1080/21505594.2017.1414134. 29252102 PMC5955196

[42] Ramirez C, Hauser AD, Vucic EA, Bar-Sagi D. 2019. Plasma membrane V-ATPase controls oncogenic RAS-induced macropinocytosis. Nature 576:477–481. doi:10.1038/s41586-019-1831-x. 31827278 PMC7048194

[43] Ratner D, Pontus M, Ornig A, Starheim KK, Marty-Roix R, Proulx MK, Goguen JD, Lien E. 2016. Manipulation of interleukin-1β and interleukin-18 production by Yersinia pestis effectors YopJ and YopM and redundant impact on virulence. J Biol Chem 291:9894–9905. doi:10.1074/jbc.M115.697698. 26884330 PMC4858993

[44] Patel JC, Galan JE. 2005. Manipulation of the host actin cytoskeleton by Salmonella—all in the name of entry. Curr Opin Microbiol 8:10–15. doi:10.1016/j.mib.2004.09.001. 15694851

[45] Müller MP, Peters H, Blümer J, Blankenfeldt W, Goody RS, Itzen A. 2010. The Legionella effector protein DrrA AMPylates the membrane traffic regulator Rab1b. Science 329:946–949. doi:10.1126/science.1192276. 20651120

[46] Neunuebel MR, Chen Y, Gaspar AH, Backlund PS, Yergey A, Machner MP. 2011. De-AMPylation of the small GTPase Rab1 by the pathogen Legionella pneumophila. Science 333:453–456. doi:10.1126/science.1207193. 21680813 PMC3209958

[47] Mukherjee S, Liu X, Arasaki K, McDonough J, Galan JE, Roy CR. 2011. Modulation of Rab GTPase function by a protein phosphocholine transferase. Nature 477:103–106. doi:10.1038/nature10335. 21822290 PMC3206611

[48] Campanacci V, Mukherjee S, Roy CR, Cherfils J. 2013. Structure of the Legionella effector AnkX reveals the mechanism of phosphocholine transfer by the FIC domain. EMBO J 32:1469–1477. doi:10.1038/emboj.2013.82. 23572077 PMC3655470

[49] Creasey EA, Isberg RR. 2012. The protein SdhA maintains the integrity of the Legionella-containing vacuole. Proc Natl Acad Sci U S A 109:3481–3486. doi:10.1073/pnas.1121286109. 22308473 PMC3295292

[50] Laguna RK, Creasey EA, Li Z, Valtz N, Isberg RR. 2006. A Legionella pneumophila-translocated substrate that is required for growth within macrophages and protection from host cell death. Proc Natl Acad Sci U S A 103:18745–18750. doi:10.1073/pnas.0609012103. 17124169 PMC1656969

[51] Tan Y, Arnold RJ, Luo ZQ. 2011. Legionella pneumophila regulates the small GTPase Rab1 activity by reversible phosphorylcholination. Proc Natl Acad Sci U S A 108:21212–21217. doi:10.1073/pnas.1114023109. 22158903 PMC3248503

[52] Isberg RR, O'Connor TJ, Heidtman M. 2009. The Legionella pneumophila replication vacuole: making a cozy niche inside host cells. Nat Rev Microbiol 7:13–24. doi:10.1038/nrmicro1967. 19011659 PMC2631402

[53] Michard C, Doublet P. 2015. Post-translational modifications are key players of the Legionella pneumophila infection strategy. Front Microbiol 6:87. doi:10.3389/fmicb.2015.00087. 25713573 PMC4322725

[54] Guo X, Dong Y, Yin S, Zhao C, Huo Y, Fan L, Hu H. 2013. Patulin induces pro-survival functions via autophagy inhibition and p62 accumulation. Cell Death Dis 4:e822. doi:10.1038/cddis.2013.349. 24091665 PMC3824659

[55] Lee YR, Kuo SH, Lin CY, Fu PJ, Lin YS, Yeh TM, Liu HS. 2018. Dengue virus-induced ER stress is required for autophagy activation, viral replication, and pathogenesis both in vitro and in vivo. Sci Rep 8:489. doi:10.1038/s41598-017-18909-3. 29323257 PMC5765116

[56] Fung TS, Liu DX. 2019. The ER stress sensor IRE1 and MAP kinase ERK modulate autophagy induction in cells infected with coronavirus infectious bronchitis virus. Virology 533:34–44. doi:10.1016/j.virol.2019.05.002. 31082732 PMC7112053

[57] Fun XH, Thibault G. 2020. Lipid bilayer stress and proteotoxic stress-induced unfolded protein response deploy divergent transcriptional and non-transcriptional programmes. Biochim Biophys Acta Mol Cell Biol Lipids 1865:158449. doi:10.1016/j.bbalip.2019.04.009. 31028913

[58] Sherman F, Fink GR, Hicks JB. 1986. Methods in yeast genetics: laboratory course manual for methods in genetics. Cold Spring Harbor Laboratory, Cold Spring Harbor, NY.

