# Supplementary figures and images for: Manipulation of IRE1-Dependent MAPK Signaling by a Vibrio Agonist-Antagonist Effector Pair
Source: mSystems. 2021 Feb 9;6(1):e00872-20. doi: 10.1128/mSystems.00872-20 (PMC7883537; doi:10.1128/mSystems.00872-20)

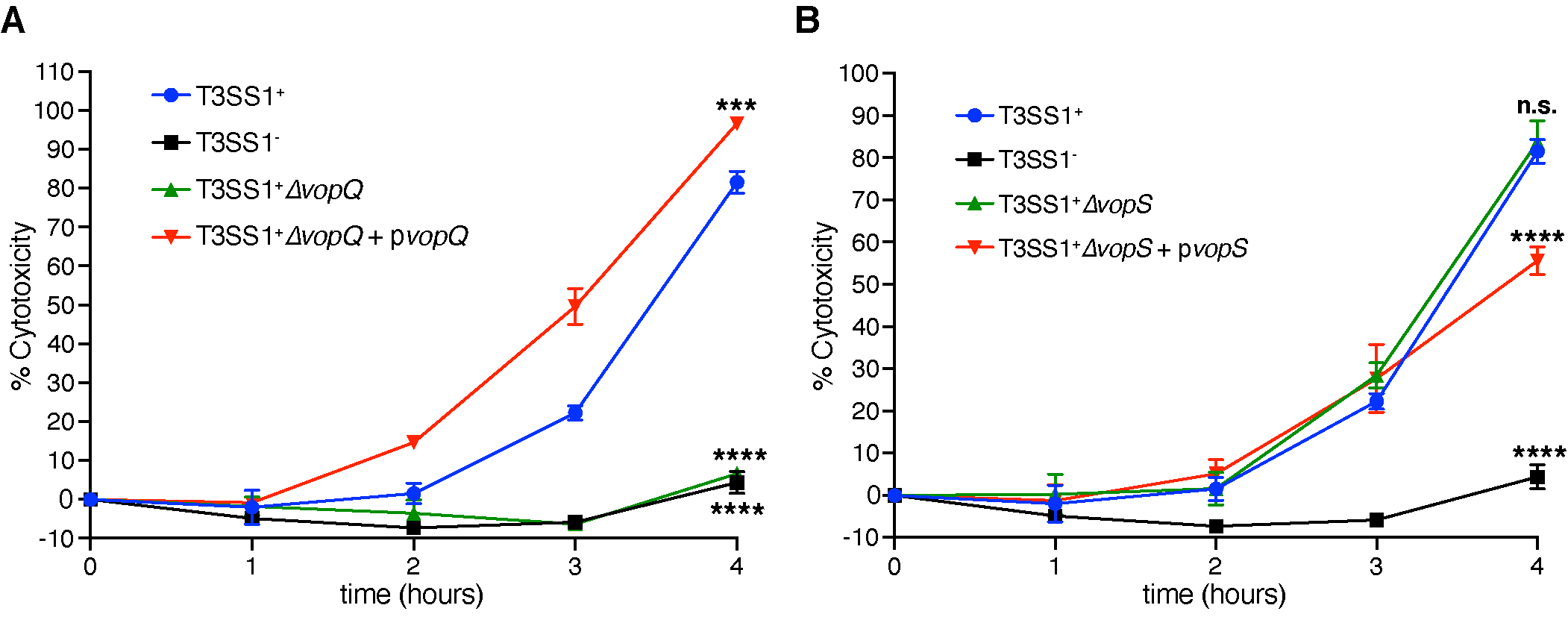

Supplement: FIG S1 [file mSystems.00872-20-sf001.tif]

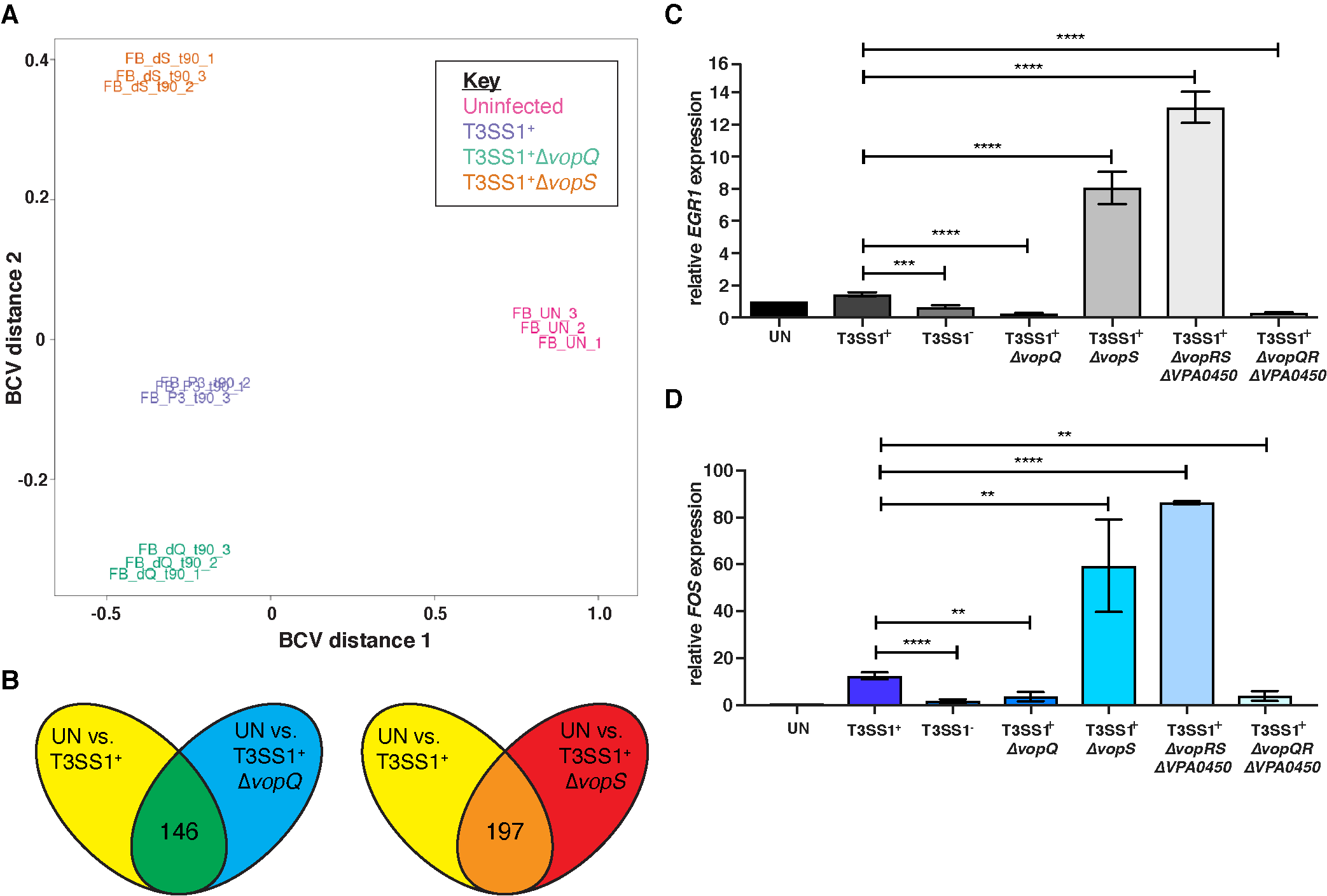

Supplement: FIG S2 [file mSystems.00872-20-sf002.tif]

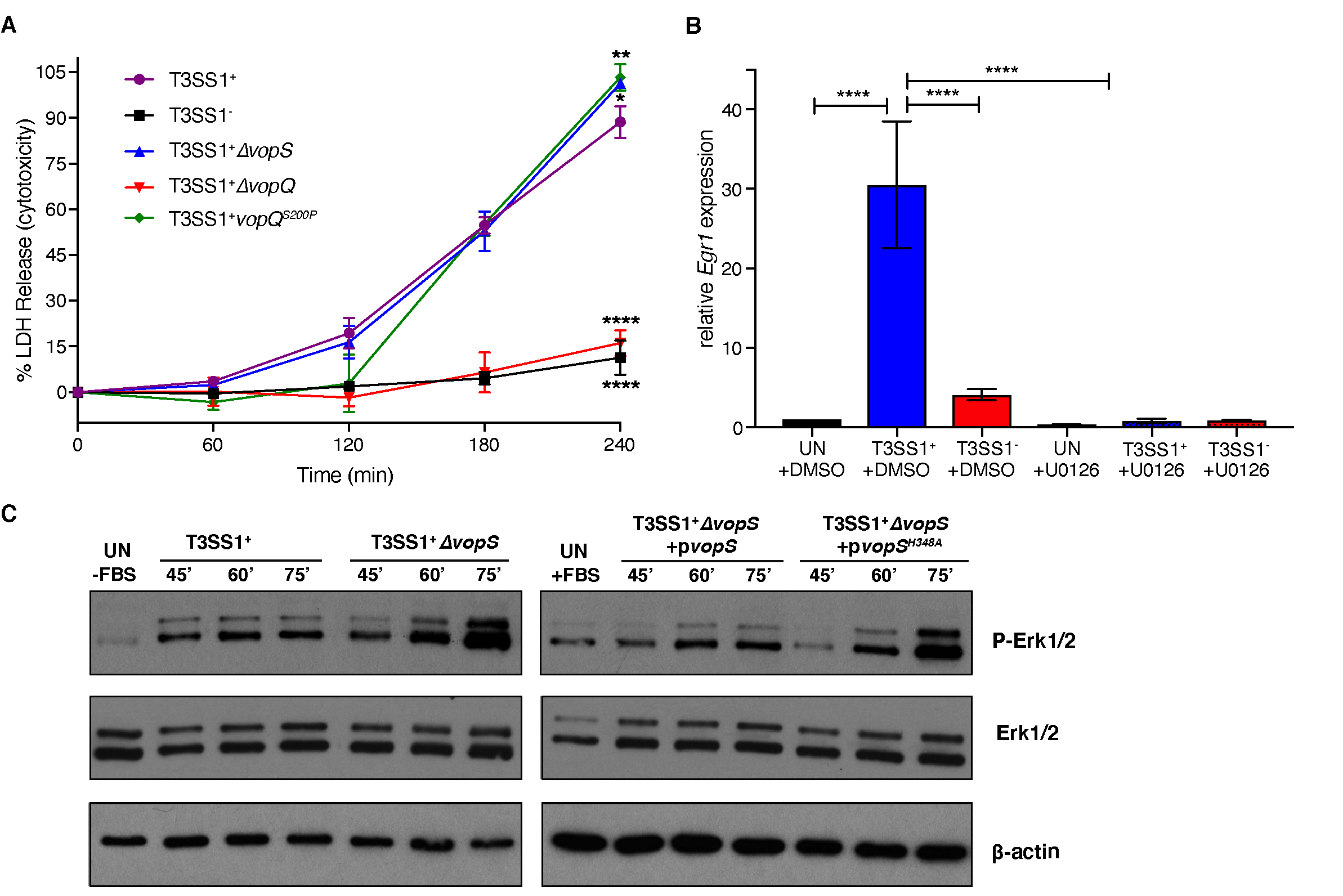

Supplement: FIG S3 [file mSystems.00872-20-sf003.tif]

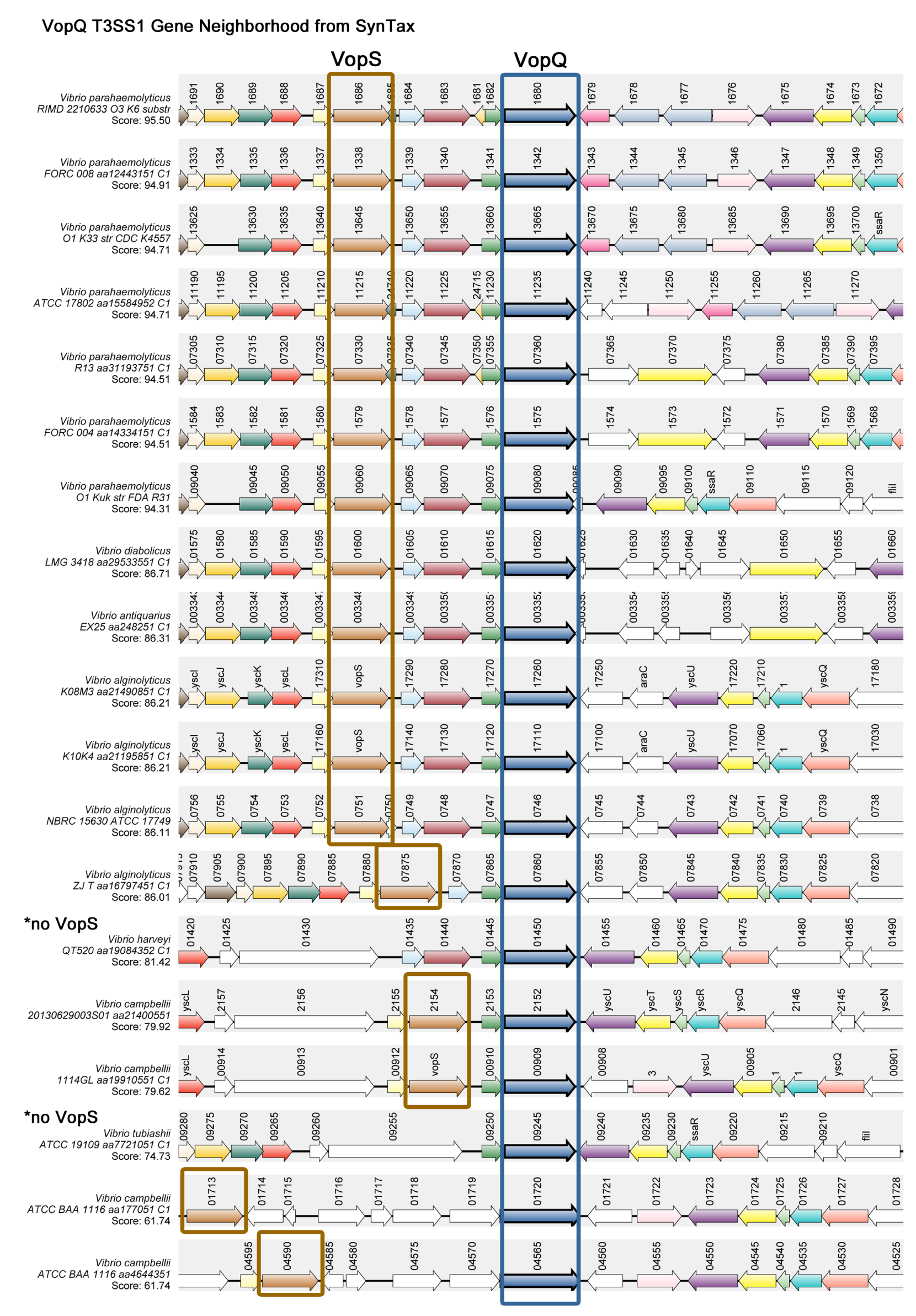

Supplement: FIG S4 [file mSystems.00872-20-sf004.tif]

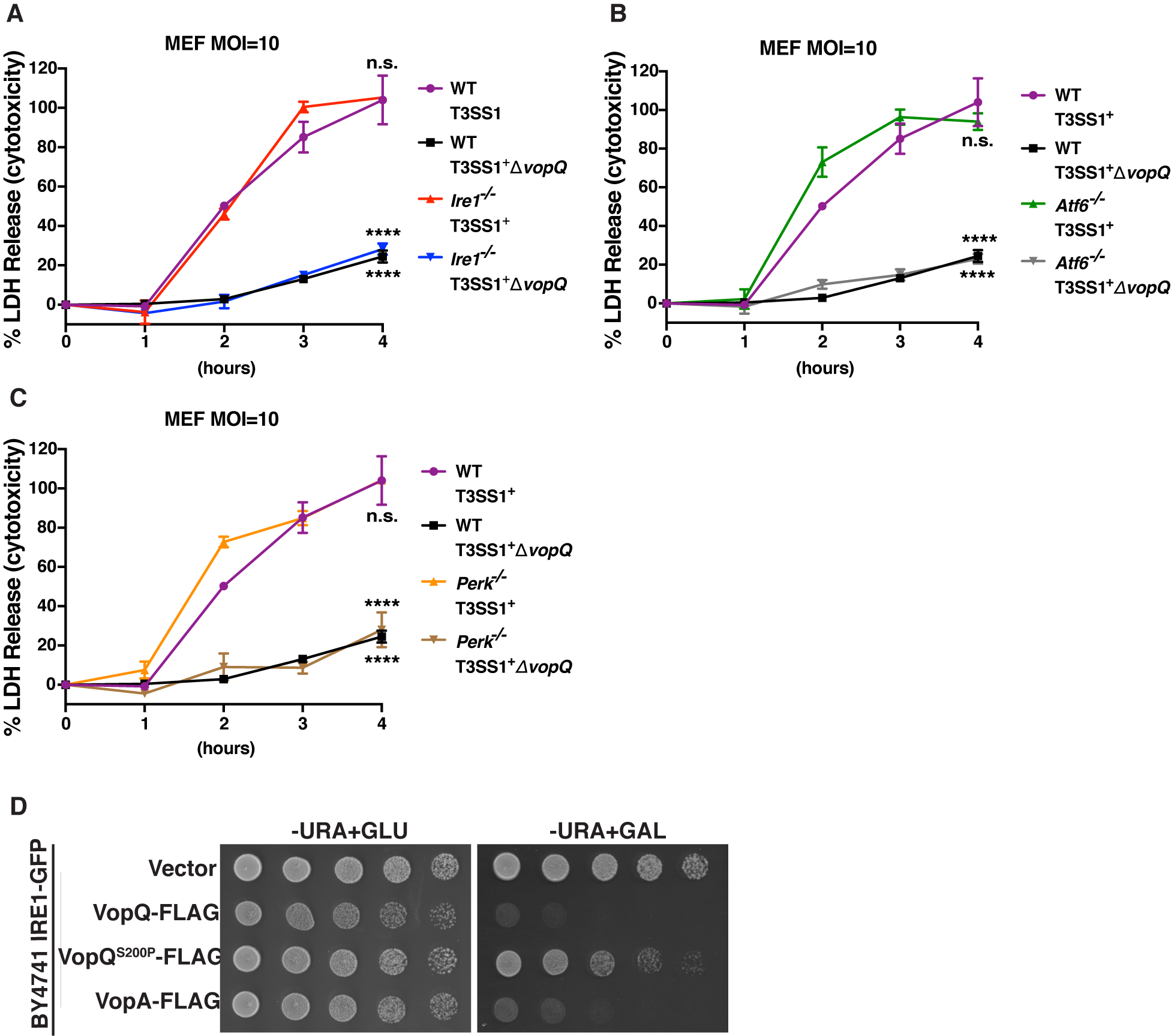

Supplement: FIG S5 [file mSystems.00872-20-sf005.tif]
