# Supplementary material for: Manipulation of IRE1-Dependent MAPK Signaling by a Vibrio Agonist-Antagonist Effector Pair
Source: mSystems. 2021 Feb 9;6(1):e00872-20. doi: 10.1128/mSystems.00872-20 (PMC7883537; doi:10.1128/mSystems.00872-20)
Supplement: TABLE S1 [file mSystems.00872-20-st001.docx]

| **Strain Name (this work)** | **Strain Name (systematic)** | **Genotype** | **Species/Strain** | **Source** |
| --- | --- | --- | --- | --- |
| T3SS1^+^ | POR3 | Δ*tdhAS*Δ*vcrD2* | *Vibrio parahaemolyticus RIMD2210633* | (*6*) |
| T3SS1^-^ | POR4 | Δ*tdhAS*Δ*vcrD1*Δ*vcrD2* | *Vibrio parahaemolyticus RIMD2210633* | (*6*) |
| T3SS1^+^Δ*vopQ* | POR3Δ*vopQ* | Δ*tdhAS*Δ*vcrD2*Δ*vp1680* | *Vibrio parahaemolyticus RIMD2210633* | (*11*) |
| T3SS1^+^Δ*vopS* | POR3Δ*vopS* | Δ*tdhAS*Δ*vcrD2*Δ*vp1686* | *Vibrio parahaemolyticus RIMD2210633* | (*8*) |
| T3SS1^+^Δ*vopQ*+p*vopQ* | POR3Δ*vopQ*+  p*vopQ* | Δ*tdhAS*Δ*vcrD2*Δ*vp1680*  pLAFR*-vopS*(Tet^R^) | *Vibrio parahaemolyticus RIMD2210633* | (*11*) |
| T3SS1^+^Δ*vopS*+p*vopS* | POR3Δ*vopQ*+  p*vopQ* | Δ*tdhAS*Δ*vcrD2*Δ*vp1686*  pLAFR*-vopS*(Tet^R^) | *Vibrio parahaemolyticus RIMD2210633* | (*8*) |
| T3SS1^+^Δ*vopRS*  Δ*VPA0450* | CAB3Δ*RS0450* | Δ*tdhAS*Δ*vtrA*Δ*vp1683*  Δ*vp1686*Δ*vpa0450* | *Vibrio parahaemolyticus RIMD2210633* | (*51*) |
| T3SS1^+^Δ*vopQR*  Δ*VPA0450* | CAB5 | Δ*tdhAS*Δ*vtrA*Δ*vp1680*  Δ*vp1683*Δ*vpa0450* | *Vibrio parahaemolyticus RIMD2210633* | (*51*) |
| T3SS1^+^*vopQ*^S200P^ | POR3::*vopQ*^S200P^ | Δ*tdhAS*Δ*vcrD2*  Δ*vp1680::vp1680*^S200P^ | *Vibrio parahaemolyticus RIMD2210633* | This work |
