# Supplementary material for: Manipulation of IRE1-Dependent MAPK Signaling by a Vibrio Agonist-Antagonist Effector Pair
Source: mSystems. 2021 Feb 9;6(1):e00872-20. doi: 10.1128/mSystems.00872-20 (PMC7883537; doi:10.1128/mSystems.00872-20)
Supplement: TABLE S2 [file mSystems.00872-20-st002.docx]

|  | T3SS1^+^ vs. T3SS1^+^ΔvopQ | | T3SS1^+^ vs. T3SS1^+^ΔvopS | | |  |
| --- | --- | --- | --- | --- | --- | --- |
| Gene | log_2_ FC | FDR | log_2_ FC | FDR | Description | |
| EGR1 | -1.29069 | 3.81x10^-46^ | 2.995356 | 3.88x10^-261^ | TF, ERK1/2 signaling target gene | |
| c-JUN | -0.55013 | 0.00678 | -0.37351 | 0.23864 | TF, JNK signaling target gene | |
| BAX | -0.05928 | 0.68302 | -0.27029 | 0.02194 | Apoptosis factor, c-JUN target | |
| BCL2 | -.006621 | 0.85026 | 0.182688 | 0.66732 | Apoptosis factor, c-JUN target | |
| PLAU | 1.16178 | 1.61x10^-62^ | 0.615908 | 1.98x10^-8^ | Protease, AP-1 complex target | |
| ATF2 | 0.01896 | 0.95446 | 0.01331 | 1 | TF, JNK signaling target gene | |
| ATF3 | -1.39532 | 2.01x10^-61^ | -0.59471 | 1.17x10^-11^ | TF, Target of ATF2+c-JUN | |
| TNF | 1.86787 | 7.23x10^-19^ | -0.00713 | 1 | Cytokine, target of ATF2+c-JUN | |
| ELK1 | -0.16185 | 0.31897 | 0.00500 | 1 | TF, JNK signaling target gene | |
| MCL1 | -0.163371 | 0.05982 | 0.65358 | 4.23x10^-23^ | Apoptosis factor, ELK1 target | |
| PDLIM5 | -0.18618 | 0.11409 | 0.10029 | 0.66732 | Scaffolding protein, ELK1 target | |
