# Supplementary material for: Manipulation of IRE1-Dependent MAPK Signaling by a Vibrio Agonist-Antagonist Effector Pair
Source: mSystems. 2021 Feb 9;6(1):e00872-20. doi: 10.1128/mSystems.00872-20 (PMC7883537; doi:10.1128/mSystems.00872-20)
Supplement: TEXT S1 [file mSystems.00872-20-s0001.docx]

­­_­­_**Title: Manipulation of noncanonical IRE1-dependent MAPK signaling by a Vibrio agonist-antagonist effector pair**

**Supplemental Materials and Methods**

*RNA-sequencing Analysis*

Fastq files were analyzed using fastqc v0.11.2(*44*) and fastq_screen v0.4.4(*45*). Reads were quality-trimmed using fastq-mcf (ea-utils/1.1.2-806)(*46*). Trimmed reads were mapped to the hg19 assembly of the human genome (UCSC version from igenomes) using TopHat(*47*) and duplicated reads marked using picard-tools (v1.127 <https://broadinstitute.github.io/picard/>). Read counts were generated using featureCounts(*48*) and differential expression (DE) analysis was performed using edgeR(*49*). Pathway and network analysis were performed with QIAGEN’s Ingenuity^®^ Pathway Analysis tool (IPA^®^, QIAGEN Redwood City, <http://www.qiagen.com/ingenuity>). DE gene heat maps were hierarchically clustered using R (<http://www.R-project.org>).

*Quantitative RT-PCR*

Primers for human and mouse *EGR1, FOS and IPO8* were previously described(*3*). RNA was isolated from infected PHDFs and MEFs 90 minutes post infection by the methods described above. RNA concentration was measured via NanoDrop and cDNA was generated using the iScript cDNA Synthesis kit (BioRad). Transcripts were quantified on a CFX384 Touch Real-Time PCR Detection System (CFX Manager™ software) using iTaq Universal SYBR Green Supermix (BioRad) and 500nM primers. Relative gene expression for each target gene was calculated by the ΔΔCq method with respect to transcript levels in uninfected cells using the reference gene *IPO8* for normalization(*50*). Statistical analysis was performed with Prism 7 software.

*Lactate dehydrogenase (LDH)-release Assay*

MEFs and PHDFs were seeded into 24-well plates at a density of 10^5^ cells/mL. Cells were washed in un-supplemented DMEM (-phenol red) prior to infection. Pre-induced *V. para* strains were added at an MOI=10 as described above. Triplicate medium samples were collected at each time point and transferred to a 96-well plate. The activity of the LDH enzyme was measured using the LDH Cytotoxicity Detection Kit (Takara-Clonetech) on a FLUOstar OPTIMA plate reader with OPTIMA software. Percent LDH-release was calculated compared to cells lysed in 1% Triton X-100.

*Co-occurrence and synteny analysis*

*Vibrio* strains that retained synteny in the T3SS1 gene neighborhood were identified using the SyntTax web server (PMID: 23323735). All strains (223 genomes) were searched using VopQ and VopS sequences as queries. Searches were performed using best match search parameters with 15% normal BLAST minimum score. Genomes with identical T3SS1 neighborhoods were filtered for Fig. S4.
